# Screening and Comprehensive Analysis of Cancer-Associated tRNA-Derived Fragments

**DOI:** 10.3389/fgene.2021.747931

**Published:** 2022-01-14

**Authors:** Yiran Zhou, Qinghua Cui, Yuan Zhou

**Affiliations:** ^1^ MOE Key Lab of Cardiovascular Sciences, Department of Biomedical Informatics, Center for Noncoding RNA Medicine, School of Basic Medical Sciences, Peking University, Beijing, China; ^2^ MOE Key Lab of Cardiovascular Sciences, Department of Physiology and Pathophysiology, Center for Noncoding RNA Medicine, School of Basic Medical Sciences, Peking University, Beijing, China

**Keywords:** tRNA-derived fragments, cancer prognosis, cancer subtype, bioinformactics analysis, cancer immunity, small non coding RNAs

## Abstract

tRNA-derived fragments (tRFs) constitute a novel class of small non-coding RNA cleaved from tRNAs. In recent years, researches have shown the regulatory roles of a few tRFs in cancers, illuminating a new direction for tRF-centric cancer researches. Nonetheless, more specific screening of tRFs related to oncogenesis pathways, cancer progression stages and cancer prognosis is continuously demanded to reveal the landscape of the cancer-associated tRFs. In this work, by combining the clinical information recorded in The Cancer Genome Atlas (TCGA) and the tRF expression profiles curated by MINTbase v2.0, we systematically screened 1,516 cancer-associated tRFs (ca-tRFs) across seven cancer types. The ca-tRF set collectively combined the differentially expressed tRFs between cancer samples and control samples, the tRFs significantly correlated with tumor stage and the tRFs significantly correlated with patient survival. By incorporating our previous tRF-target dataset, we found the ca-tRFs tend to target cancer-associated genes and onco-pathways like ATF6-mediated unfolded protein response, angiogenesis, cell cycle process regulation, focal adhesion, PI3K-Akt signaling pathway, cellular senescence and FoxO signaling pathway across multiple cancer types. And cell composition analysis implies that the expressions of ca-tRFs are more likely to be correlated with T-cell infiltration. We also found the ca-tRF expression pattern is informative to prognosis, suggesting plausible tRF-based cancer subtypes. Together, our systematic analysis demonstrates the potentially extensive involvements of tRFs in cancers, and provides a reasonable list of cancer-associated tRFs for further investigations.

## Introduction

As its name implied, tRNA-derived fragment (tRF), is a novel class of non-coding RNA (ncRNA) cleaved from mature transfer RNAs (tRNA) (Lee et al., 2009; Thompson and Parker, 2009). In early days, tRFs were widely misunderstood as tRNA degeneration byproducts. However, in recent years, extending scope of tRFs’ biological functions had been uncovered, bringing tRFs back to researchers’ view (Li et al., 2018). For example, tRFs can be loaded onto Argonaute (AGO) family proteins to perform microRNA-like post-transcriptional regulations on target RNAs (Li et al., 2012; Shao et al., 2017). Some tRFs are also found capable of facilitating ribosome biogenesis by interacting with Twi12 to enhance pre-rRNA processing (Couvillion et al., 2012) or accelerating the mRNA translation of ribosomal proteins (Kim et al., 2017). Besides, some other tRFs were also reported to be capable to reduce global translation efficiency (Yamasaki et al., 2009; Ivanov et al., 2011), regulate immuno-functions (Wang et al., 2006) and serve as epigenetic regulators (Chen et al., 2016).

Comparable to tRFs’ functions, their dysregulation could be associated with various diseases such as nonalcoholic fatty liver disease (Zhu et al., 2020), Alzheimer’s disease (Wu et al., 2021), arterial injury (Zhu et al., 2021) and especially cancers (Balatti et al., 2017; Shao et al., 2017; Falconi et al., 2019; Zhu et al., 2019; Gu et al., 2020; Zhang et al., 2021). Especially, a series of investigations have uncovered novel associations between tRFs and cancers. For instance, Falconi et al. found that a new tRF derived from the 3′-end of tRNA-Glu is significantly down-regulated in breast cancer, and finally validated its tumor repressive role (Falconi et al., 2019). Zhu et al. compared the plasma tRNA levels between liver cancer patients and healthy donors, and rationally determined four tRFs as diagnostic biomarkers (Zhu et al., 2019). However, in most of the above experimental researches on tRF-cancer associations, only few tRFs and cancer types were considered due to the limitation of low-throughput approaches or sample size. Therefore, specific computational screening of cancer-associated tRFs based on high-throughput datasets is continuously in demand to better understand the roles of tRFs in cancer, including but not limited to tRF dysregulation in various cancer types, key oncogenesis pathways targeted by tRFs and the associations between tRF expression pattern and cancer progression and prognosis.

Recently, Rigoutsos lab established a comprehensive database for human tRFs termed MINTbase v2.0 (Pliatsika et al., 2018), which provides detailed annotations of 26,744 tRFs. More importantly, by re-analyzing the small RNA-sequencing library from TCGA project (Cancer Genome Atlas Research et al., 2013), MINTbase v2.0 also provides 10,814 tRF expression profiles. This dataset provides an unprecedented chance for extensive investigating the characteristics of tRFs in cancer. Indeed, based on this dataset, Rigoutsos lab has revealed a lot of biological characteristics of tRFs in various cancer types. For example, in 2015 they firstly investigated the tRF length distribution in the breast cancer dataset and revealed the tRF expression dependence on race (Telonis et al., 2015). Subsequently in 2018, The tRF profiles of prostate cancer were also uncovered (Magee et al., 2018). In the same year, they constructed a complex tRF-miRNA-mRNA co-expression network for triple-negative breast cancer and then discovered many altered mRNA-mRNA co-expression associations depending on disease state and race. More importantly, this heterogeneity could be largely explained by differential tRF-mRNA co-expression, demonstrating tRFs’ important biological functions (Telonis and Rigoutsos, 2018). The investigation of tRF-mRNA co-expression network was soon extended to a pan-cancer scale (32 cancer types) (Telonis et al., 2019), where they identified more tRF-involved pathways and additionally found some pathways are regulated by tRFs in a sex-dependent manner, further highlighting the regulatory roles of tRFs. However, on the other hand, their researches tended to depict the general biological characteristics of tRFs (for example, tRF length variation, sex- and race-dependent disparity and tRF-mRNA co-expression pattern) on the whole set of widely-expressed tRFs, but not focused on the specific screening about tRFs that are associated with oncogenesis, cancer progression and prognosis. Therefore, it is still necessary to investigate the roles of tRFs in cancer by a more specifically designed computational pipeline that combines tRF expression with available clinical data of cancer patients. In this work, based on the annotations from MINTbase v2.0 and the clinical information of TCGA samples, we firstly screened cancer-associated tRFs (ca-tRFs) across seven TCGA cancer types, including breast invasive carcinoma (BRCA), head and neck squamous cell carcinoma (HNSC), kidney renal clear cell carcinoma (KIRC), kidney renal papillary cell carcinoma (KIRP), liver hepatocellular carcinoma (LIHC), lung squamous cell carcinoma (LUSC) and thyroid carcinoma (THCA). Then, a series of analyses were conducted to define the functional characteristics of ca-tRFs. In the following text, we will firstly describe our integrative analysis pipeline, and then the analysis results and discussion thereof.

## Materials and Methods

### Screening of ca-tRFs

**FIGURE 1:**
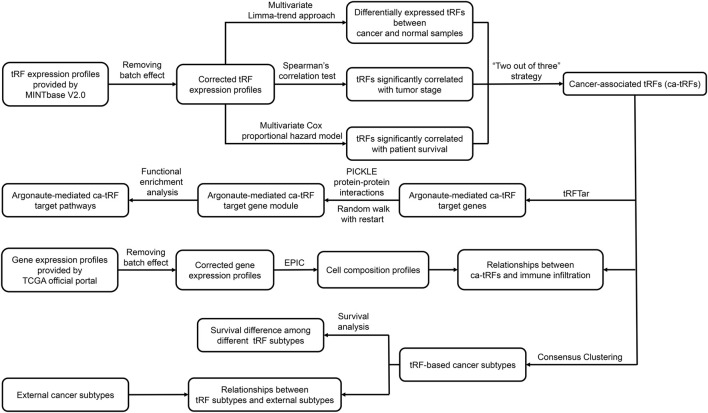
Analysis pipeline of the work. In this work, based on the tRF expression profiles of TCGA samples provided by MINTbase V2.0, we screened cancer-associated tRFs (ca-tRF) for seven cancer types, including BRCA, HNSC, KIRC, KIRP, LIHC, LUSC, and THCA, by considering three candidate tRF sets including the differentially expressed tRFs between cancer samples and normal samples (cde-tRF), the tRFs significantly correlated with clinical tumor stage (tsca-tRF) and the tRFs significantly correlated with patient survival (psca-tRF). The ca-tRFs were further determined by a “two out of three strategy,” which means a ca-tRF must belong to at least two of the total three sets. Then we performed extensive analyses on the screened ca-tRF set. First, by incorporating the dataset of Argonaute-mediated tRF-target gene interactions from the tRFTar database, we located the ca-tRF target genes and further the target gene modules. Then by performing functional enrichment analysis on the gene modules, the carcinogenic and cancer-promoting roles of ca-tRFs were inferred. Subsequently, we deconvoluted the gene profiles of TCGA samples *via* the EPIC tool to measure the immune infiltration degree and further demonstrated the immuno-regulatory roles of ca-tRFs. Next, by means of consensus clustering, we identified several ca-tRF-based cancer subtypes. Survival curves exhibit significant survival differences among these subtypes, indicating the prognostic values of ca-tRFs. Finally, to reveal the dependence or independence of ca-tRFs on the other molecular signatures, the relations between ca-tRF subtypes and various existing external subtypes were investigated.

The overall computational pipeline of this work is depicted in Figure 1. Datasets and key source code used in this work were also uploaded to the GitHub (https://github.com/Load-Star/ca-tRF/). In this work, we screened ca-tRFs by integrating the tRF expression profiles and the clinical information of the TCGA samples, which were obtained from the MINTbase V2.0 (Pliatsika et al., 2018) database and the TCGA official portal (Cancer Genome Atlas Research et al., 2013), respectively. To adapt downstream analyses, the tRF expression data was log2-transformed and processed with batch effect correction by R package *limma* (Ritchie et al., 2015), where the tissue source site and plate ID of each sample were modeled as the correction covariates. In the following step for ca-tRF screening, we considered three candidate tRF sources: 1) the differentially expressed tRFs between cancer samples and normal samples (referred as cde-tRF hereafter) identified by multivariate limma-trend approach (by R package *limma*), where the tissue type (cancer or normal), patient sex and race were modeled as dummy variables while patient age was modeled as numeric variable; 2) the tRFs significantly correlated with clinical tumor stage (referred as tsca-tRF hereafter) identified by Spearman’s rank correlation test (by python package *Scipy*); 3) the tRFs significantly correlated with patient survival (referred as psca-tRF hereafter) detected by multivariate Cox proportional hazards model (by python package *lifelines*), where the patient sex and race were modeled as dummy variables while tRF expression abundance and patient age were modeled as numeric variables. The *p*-values were corrected by Storey’s q-value approach. To ensure the robustness of the results, here we only considered the cancer types with sufficient samples and clinical information about tumor stage and survival, including BRCA, HNSC, KIRC, KIRP, LIHC, LUSC, and THCA. Besides, only the tRFs detectable (with an expression threshold of no less than 1 RPM) in no less than 10% samples were retained. In addition, we applied a “two out of three” strategy for further false positive control. More specifically, only if one tRF passed at least two of the total three tests (namely the above-mentioned multivariate limma-trend approach, Spearman’s rank correlation test and multivariate Cox proportional hazards model), it could be listed as a ca-tRF of the corresponding cancer type. According to our observations, the amounts of tRFs overrepresented by Spearman’s correlation test and Cox regression model are much smaller than those overrepresented by limma-trend approach. Therefore, to screen enough ca-tRFs for downstream analyses, a relaxed but acceptable q-value cutoff 0.1 was used during the ca-tRF screening. The integrated list of screened ca-tRFs were shown in Supplementary Data S1.

### Identification and Functional Investigation of Gene Modules Targeted by ca-tRFs

Like microRNAs, some tRFs are also capable of binding AGO-family proteins and regulate target genes. Recently, we identified a considerable number of tRFs and genes from AGO-associated crosslinking-immunoprecipitation and high-throughput sequencing (AGO-CLIP) libraries and strictly screened AGO-mediated tRF-gene interactions by computationally simulating the base pairing (more specifically, annealing processes) between tRFs and the regions in mRNAs/lncRNAs covered by AGO-CLIP. The interactions satisfying tight base pairing (normalized free energy less than −1.25 kcal/mol) had been deposited into a database named tRFTar (Zhou et al., 2021). Here in order to understand the biological functions that the ca-tRFs may participate in, ca-tRF target genes were retrieved from tRFTar. To control the false positive rate, only the interactions supported by tRF-gene co-expression in TCGA samples were retained. Our hypothesis is that tRFs that 1) show tight base-pairing with potential target genes on Argonaute-covered target regions and 2) show significant expression correlation with potential target genes potentially act as regulatory targeting factors. It is also likely that tRFs themselves could be served as the targets of other regulatory molecules like miRNAs but we here only focused on tRF-gene interactions to avoid much complicated reasoning that involves tRF-miRNA-gene triplexes. Then for each cancer type, taking the ca-tRF target genes as seed genes, an extended gene module targeted by ca-tRFs was detected by the algorithm of random walk with restart (RWR) on the protein-protein interaction (PPI) network. The PPI network was constructed from 214,666 cross-validated interactions in the PICKLE 3.0 database (Dimitrakopoulos et al., 2020). The RWR algorithm could be described as the following steps:

(1) Construct an adjacent square matrix *A* to represent whether the gene *i* (the *i-*th row) and gene *j* (the *j-*th column) are directly adjacent in the network:

$$A_{i,j}=A_{j,i}=\left\{ \begin{array}{l} 1,\;gene\;i\in \mathrm{Neighbor}\left(\mathrm{gene j}\right)\;\\ 0,else \end{array}\right.$$ (1)

where *Neighbor(gene j)* represents the gene set directly adjacent to gene *j* in the network, including gene *j* itself.

(2) Construct a transition matrix *T* to represent the probabilities from gene *i* (the *i-*th row) to gene *j* (the *j-*th column) in an iteration:

$$T_{i,j}=\frac{A_{i,j}}{\sum_{k}^{n_{gene}}A_{i,k}}$$ (2)

where *n*_*gene*_ represents the total number of genes in the network.

(3) Construct a column vector *e* as the input to represent the initial probabilities of seed genes (ca-tRF target genes here):

$$e_{i}=\{\begin{array}{ccc} 1/n_{\mathrm{seed}} & , & \mathrm{gene}\;i\in \left\{\mathrm{seed}\;\mathrm{genes}\right\}\\ 0 & , & \mathrm{else} \end{array}$$ (3)

where *n*_*seed*_ represents the total number of input seed genes.

(4) Perform iterations according to the following formula until the *s* achieves convergent:

$$s^{n+1}=\{\begin{array}{ccc} e & , & n=1\\ c\cdot e+\left(1-c\right)\cdot \left(T^{T}s^{n}\right) & , & n\neq 1 \end{array}$$ (4)

where *n* represents the iteration number and *c* is exactly the restart probability. If *c* = 1, then the *s* will be constantly equal to *e*; if *c* = 0, then the RWR model will reduce to traditional random walk model. In this work, a moderate *c* = 0.5 was selected to balance the local properties of the input and the global properties of the network.

(5) Determine the genes with top probabilities in the convergent *s* for downstream analyses, here we selected the top genes whose cumulative probability reach at 0.25 as the resulting gene module.

Next, we conducted gene functional enrichment analyses (by Fisher’s exact test) for the gene modules so that potential functional roles of ca-tRFs playing in cancers could be inferred. Firstly, according to a list of 711 cancer-associated genes provided by the NCG v6.0 database (Repana et al., 2019), we evaluated the enrichment degree of cancer-associated genes in the modules. Then, the R software package *clusterProfiler* (Yu et al., 2012) was used to perform enrichment analyses of Gene Ontology Biological Process (GO-BP) and Kyoto Encyclopedia of Genes and Genomes (KEGG) to investigate the biological processes and pathways that the gene modules are involved in. After semantic deduplication, the terms with q-value no more than 0.05 were deemed as the statistically significant results.

### Estimation of Immune Infiltration by Gene Expression Deconvolution

We downloaded tumor samples’ gene expression data from the TCGA official portal and then deconvolute them into quantitative cell composition lists by the EPIC tool (Racle et al., 2017). EPIC supposes different cell types prominently express their own cell type marker genes. Given the built-in marker genes’ abundance of several pre-defined cell types (T cell, B cell, macrophage, natural killer cell, cancer-associated fibroblast and endothelial cell), the absolute proportions of these cell types inherent in the inputted TCGA bulk gene expression profiles can be solved by constrained linear model. Here the immune infiltration degree was quantified by the proportions of T cells.

### Identification and Analysis of ca-tRF-Based Cancer Subtypes

By using the consensus clustering computational framework provided by the R package *ConsensusClusterPlus* (Wilkerson and Hayes, 2010), we clustered tumor samples’ ca-tRF expression profiles and assigned subtype labels to each tumor sample according to the clustering result (Supplementary Data S2). Consensus clustering is a widely used algorithm in biological clustering problems (Niu et al., 2016; Lu and Leong, 2018; Zhang et al., 2018). For a consensus clustering task, a basic clustering method needs to be assigned and here the PAM clustering algorithm, an improved version of K-mean clustering which is more robust to noises and outliers, was adopted. The principle of consensus clustering is measuring the distances within every sample-sample pairs by their probabilities of being clustered into the same group (called “consensus index” below) in several times of sub-sampling processes. Specifically, a sub-sampling proportion *p* (0.8 here) and times *T* (100 here) are required to be pre-defined. Then for each time, a subset of samples will be randomly selected according to *p* and then clustered by the PAM clustering algorithm. The sub-sampling process disturbs the original data structure to an extent, thus if two samples are objectively similar, they will resist this disturbance and present a high consensus index. On the contrary, two dissimilar samples will exhibit low consensus indexes. Therefore, if the clustering performed well, the overall consensus indexes of sample-sample pairs will be concentrated at 0 (for samples belonging to different groups) or 1 (for samples belonging to the same group). According to this property, we rationally determined the cluster number *k* for each cancer, namely the number of ca-tRF-based subtypes, where a smaller slope of the cumulative distribution curve between the interval of (0.2, 0.8) was preferred. In case the slopes are comparable among different *k* choices, the area under the cumulative distribution curve, which is usually approximating to the maximum when the *k* saturated, was considered as the secondary metric for the optimization of *k*.

After clustering, the timeline-dependent survival rates of distinct subtypes were visualized by Kaplan-Meier plot for each cancer type using the R packages *survival* and *survminer*. Meanwhile the overall and specific inter-subtype survival differences were evaluated by log-rank test (Supplementary Data S3). For KIRC, KIRP, and LIHC, we also collected previous subtyping results based on the other indicators such as mRNA, microRNA, methylation level and so on (Supplementary Data S4) (Cancer Genome Atlas Research Network. Electronic address and Cancer Genome Atlas Research, 2017; Ricketts et al., 2018) to check their accordance and discrepancy with ca-tRF-based subtypes. The overlapping significance between ca-tRF subtypes and the other subtypes were measured by Fisher’s exact test. We further calculated enrichment scores (ES) for LIHC samples in 236 biological pathways from KEGG and Molecular Signatures Database Hallmark (MSigDB Hallmark) (Supplementary Data S5), by single sample gene set enrichment analysis (ssGSEA) (Barbie et al., 2009). The ESs among different cancer subtypes were compared by Kruskal-Wallis test (for multi-group comparison) or Mann-Whitney test (for two-group comparison) to measure the differences of pathway activities. For all above statistical tests, a *p*-value cutoff 0.05 was used.

## Results

### Overview of ca-tRFs Across Seven TCGA Cancer Types

**FIGURE 2:**
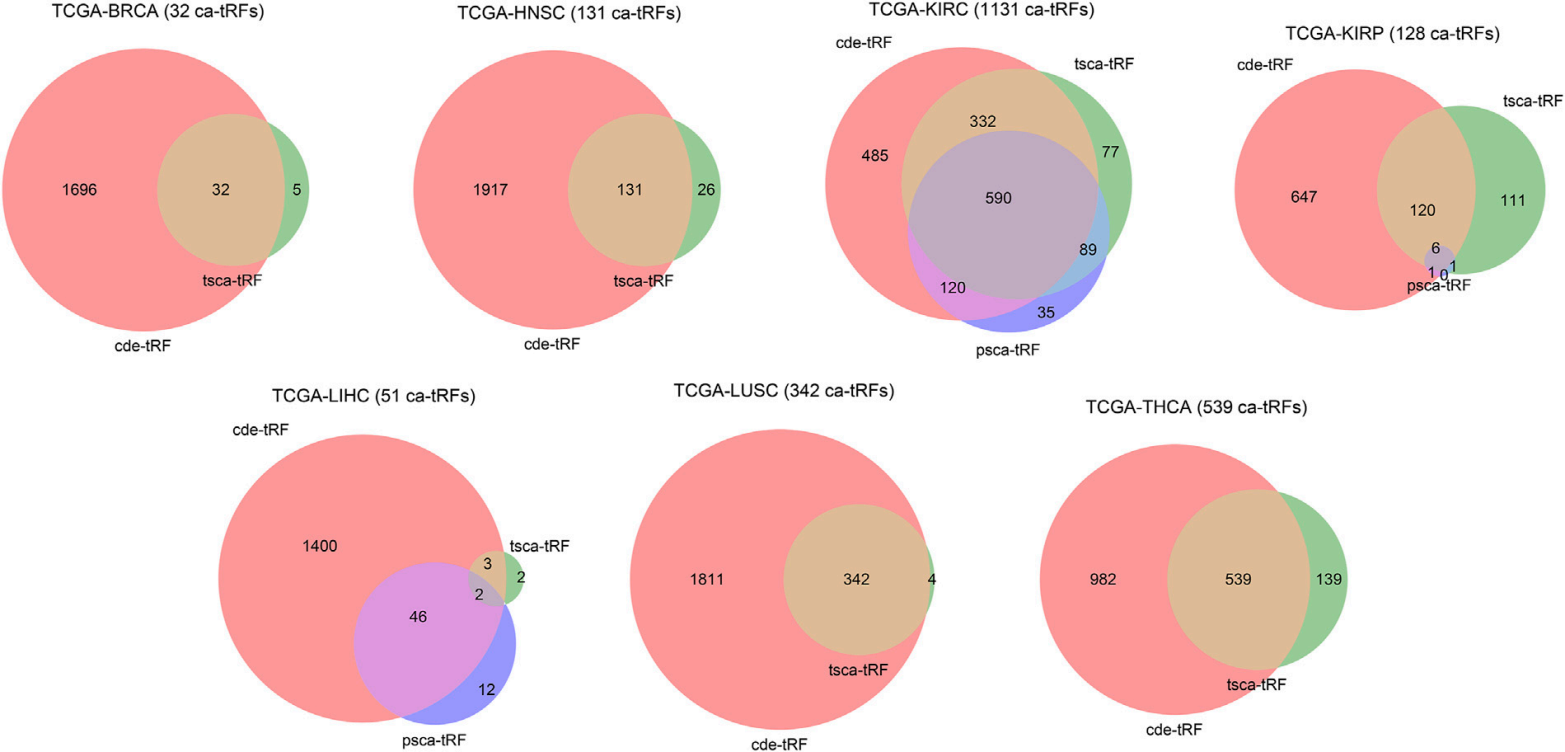
Venn diagram illustrating the intersections of three candidate ca-tRF sources. Three candidate ca-tRF sources, including the differentially expressed tRFs between cancer samples and normal samples (cde-tRF), the tRFs significantly correlated with clinical tumor stage (tsca-tRF) and the tRFs significantly correlated with patient survival (psca-tRF) were considered. For each cancer, the tRF numbers of these three sets and their intersections are shown by Venn diagram. If one tRF belongs to at least two sets (i.e., appears in any intersection area), then it was listed as a ca-tRF of the corresponding cancer. The finally determined numbers of ca-tRFs are shown following the cancer names.

**FIGURE 3:**
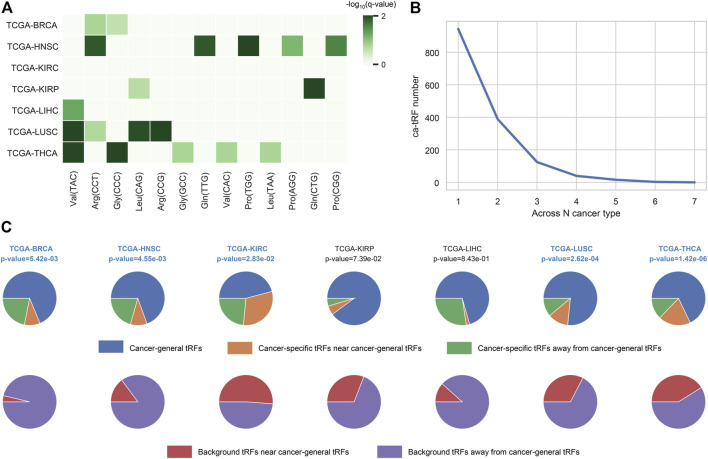
Overview of ca-tRF distribution. **(A)** The major parental tRNA isoacceptors of ca-tRFs of each cancer type. **(B)** The distribution of associated cancer type number among ca-tRFs. **(C)** The proportions of the cancer-general ca-tRFs (blue), the cancer-specific ca-tRFs near the general ca-tRFs (yellow) and the cancer-specific ca-tRFs away from the general ca-tRFs (blue). For comparison, the pie charts depicting the proportions of background tRFs near (red) and far away from (purple) the general ca-tRFs are also shown below. Here the “near” and “away from” are defined by whether the two compared tRFs are staggered with each other no more than or more than 2 nt in their parental tRNA, respectively. The statistical significance about whether the cancer-specific ca-tRFs are tending near to the general ca-tRFs than the background was measured by Fisher’s exact test and the significant cancer types were highlighted with blue and bold font.

In this work, we screened ca-tRFs for seven TCGA cancer types with sufficient samples and clinical information, including BRCA, HNSC, KIRC, KIRP, LIHC, LUSC, and THCA. Three tRF sets, including the differentially expressed tRFs between cancer samples and normal samples (cde-tRF), the tRFs significantly correlated with clinical tumor stage (tsca-tRF) and the tRFs significantly correlated with patient survival (psca-tRF), were firstly constructed as candidate sources of ca-tRFs (See Materials and Methods). If one tRF is detectable (no less than 1 RPM) in no less than 10% samples and exists in at least two sets, then it was determined as a ca-tRF (Figure 2; Supplementary Data S1). We firstly investigated the major parental tRNA isoacceptors from which these ca-tRFs were derived by using Fisher’s exact test. As the result, the ca-tRFs are found widely originated from the tRNA Val (TAC) and Arg (CCT) while the tRNA Leu (CAG) and Gly (CCC) could serve as secondary sources of ca-tRFs (Figure 3A). Then we defined cancer-specific tRFs and cancer-general tRFs as the ca-tRFs identified in only one cancer and those identified in no less than two cancers, respectively (Figure 3B). For all cancer types, a considerable proportion of cancer-general tRFs were observed. For comparison purpose, we also defined the tRFs staggered no more than 2 nt with the general ca-tRFs in their parental tRNAs as the tRFs near cancer-general tRFs. In the same way, tRFs staggered more than 2 nt with the general ca-tRFs in their parental tRNAs were defined as the tRFs far away from the cancer-general tRFs. By procedure, we found that for most cancers the cancer-specific tRFs significantly prone to be derived near the general ones in comparison with background (Fisher’s exact test; Figure 3C), outlining the sequence similarity and therefore the potential functional similarity between cancer-specific and cancer-general tRFs. Inspired by this point, we visualized the cleavage site distribution of tRFs in mature tRNAs (Supplementary Figure S1). Firstly, the background tRFs sourced from the middle or middle-latter sections of tRNAs are relatively less than those from the other sections, which is accordant with previous reports (Telonis et al., 2019). Further, in comparison with the background, the ca-tRFs of BRCA, HNSC, KIRP are found more likely from the 5′ half parts of mature tRNAs whereas the ca-tRFs of LUSC and LIHC prone to come from the 3′ half parts. That is to say, the cancer-associated tRFs may exhibit more prominent location bias in comparison with background tRFs for several cancer types.

**FIGURE 4:**
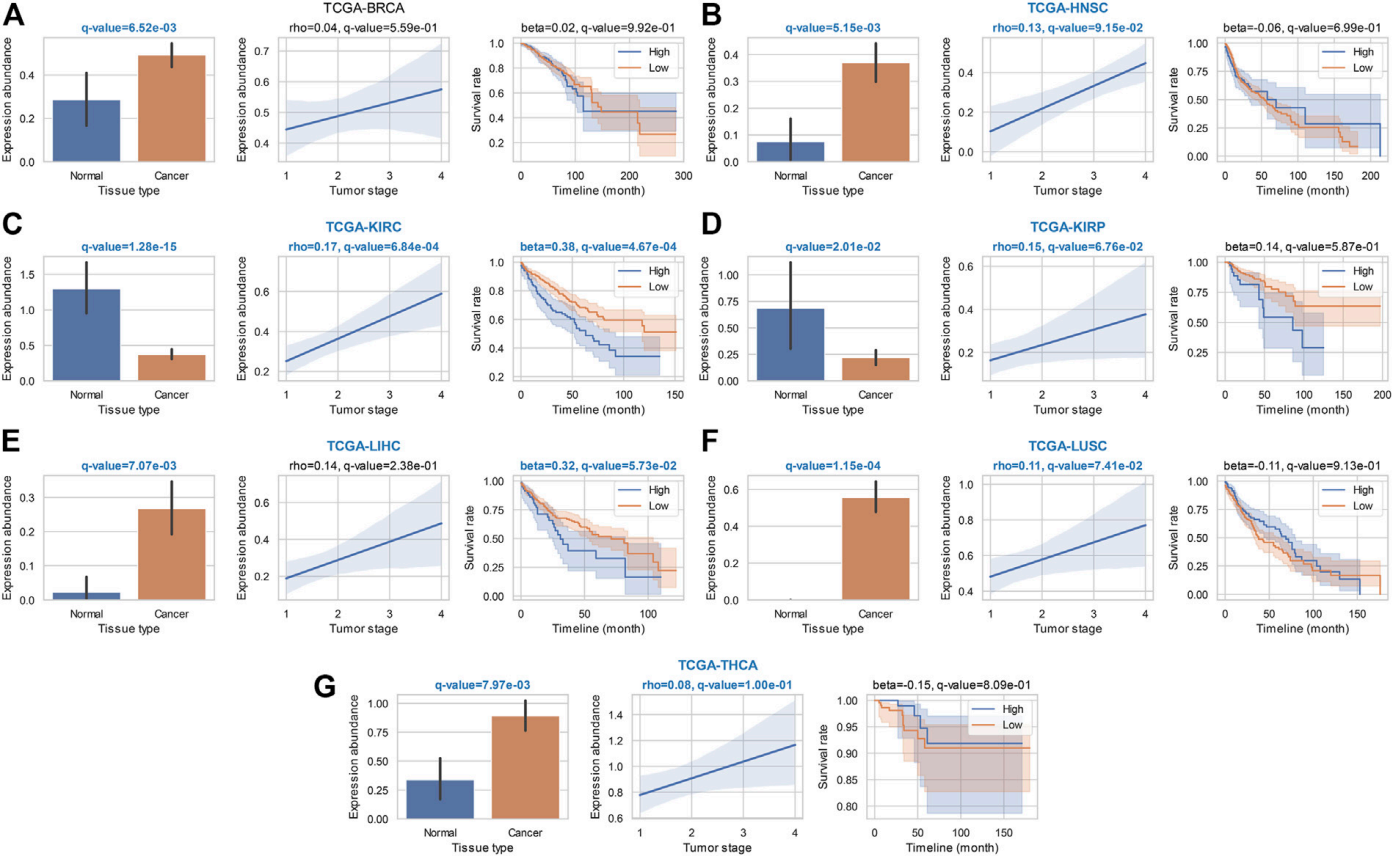
Statistical results of a widely identified ca-tRF (tRF-28-RS9NS334L2DB) in ca-tRF screening. Bar plot (with error bars), correlogram, Kaplan-Meier plot for tRF-28-RS9NS334L2DB in the statistical tests of cde-tRF, tsca-tRF, and psca-tRF screening are respectively depicted and q-values as well as key indicators (i.e., rho of Spearman’s correlation and beta of Cox regression) are shown above the plots. The significant q-values (<0.1) are highlighted with blue and bold font. Meanwhile, if tRF-28-RS9NS334L2DB is listed as ca-tRF in one cancer type (two out of three sets), the cancer type name is also highlighted. The results in **(A)** BRCA; **(B)** HNSC; **(C)** KIRC; **(D)** KIRP; **(E)** LIHC; **(F)** LUSC, and **(G)** THCA are shown accordingly.

We also noticed 19 most widely identified general ca-tRFs that are presented in at least five cancer types (Supplementary Data S1). As an instance, the statistical results of tRF-28-RS9NS334L2DB, a ca-tRF across six cancer types, are depicted in detail in Figure 4. Specifically, tRF-28-RS9NS334L2DB is significantly differential expressed in all considered seven cancer types when comparing cancer samples with normal control, and in most cancers (i.e., BRCA, HNSC, LIHC, LUSC, and THCA), an up-regulation was observed (bar plots in Figure 4). tRF-28-RS9NS334L2DB expression abundance was also found significantly positively correlated with clinical tumor stage in 5 cancers (HNSC, KIRC, KIRP, LUSC and THCA; correlograms in Figure 4) and poor prognosis in 2 cancers (KIRC and LIHC; Kaplan-Meier plots in Figure 4). Therefore, tRF-28-RS9NS334L2DB may be positively indicative in a noticeable range of cancer onset and development.

### ca-tRFs Tend to Target Cancer-Related Pathways and Associate With Tumor Immune Infiltration

**FIGURE 5:**
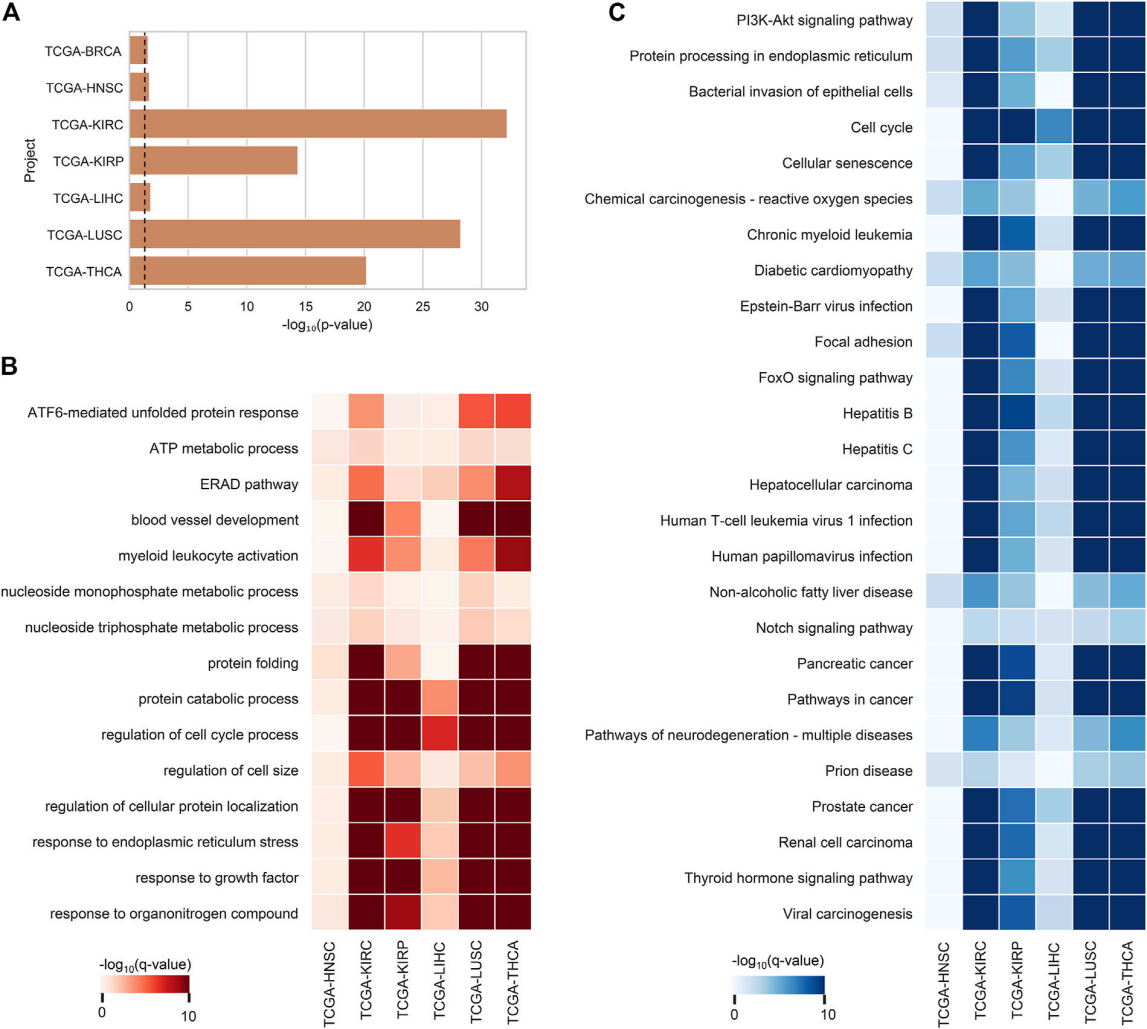
Enrichment analysis results of ca-tRF target gene modules. **(A)** The enrichment degree of cancer-associated genes (curated by NCG v6.0 database) in ca-tRF target gene modules. The dashed line represents the statistically significant cutoff (*p*-value < 0.05). **(B)** The GO-BP enrichment results of tRF target gene modules. Only the significant results (q-value < 0.05) are colored and only the terms presented in no less than 6 cancer types are depicted. **(C)** The KEGG enrichment results of tRF target gene modules. Only the significant results (q-value < 0.05) are colored and only the terms presented in no less than 5 cancer types are depicted. BRCA is not shown in subplot B and C because there are no significant terms.

After the ca-tRF screening, we further investigated their potential functions. In consideration of tRFs’ microRNA-like, AGO-dependent mRNA targeting ability, we firstly asked whether the target genes of ca-tRFs could participate in important biological processes related to cancer. According to the AGO-mediated tRF-gene interactions recorded in the tRFTar database (Zhou et al., 2021), for each cancer type we detected a significant gene module targeted by ca-tRFs by means of the RWR model on the PPI network (See Materials and Methods). We first noted that the ca-tRF target modules are enriched with the curated cancer-associated genes (Figure 5A), demonstrating the dysregulation of ca-tRFs is likely to contribute to oncogenesis and cancer development. Subsequently, referring to the gene sets of GO-BP and KEGG, we performed functional enrichment analysis for each ca-tRF target module (Figure 5B–C). Extensive terms well-known to be associated with cancer such as ATF6-mediated unfolded protein response (Lin et al., 2021), blood vessel development (Carmeliet and Jain, 2011), regulation of cell cycle process (Evan and Vousden, 2001), focal adhesion (Eke and Cordes, 2015), PI3K-Akt signaling pathway (Martini et al., 2014), cellular senescence (Campisi, 2013) and FoxO signaling pathway (Farhan et al., 2017) are presented across multi-cancer types, further supporting the associations of ca-tRFs with the cancer-related functions. It should be noted that among these terms, blood vessel development, regulation of cell cycle process, focal adhesion and PI3K-Akt signaling pathway are also identified by Telonis et al. as the pathways universally regulated by tRFs (Telonis et al., 2019). But on the other hand, there are still novel tRF target pathways that are not previously overrepresented such as ATF6-mediated unfolded protein response, cellular senescence and FoxO signaling pathway. This result also highlights the importance of specific screening the pathways that are likely disturbed by tRFs through the extensive tRF-target analysis.

**FIGURE 6:**
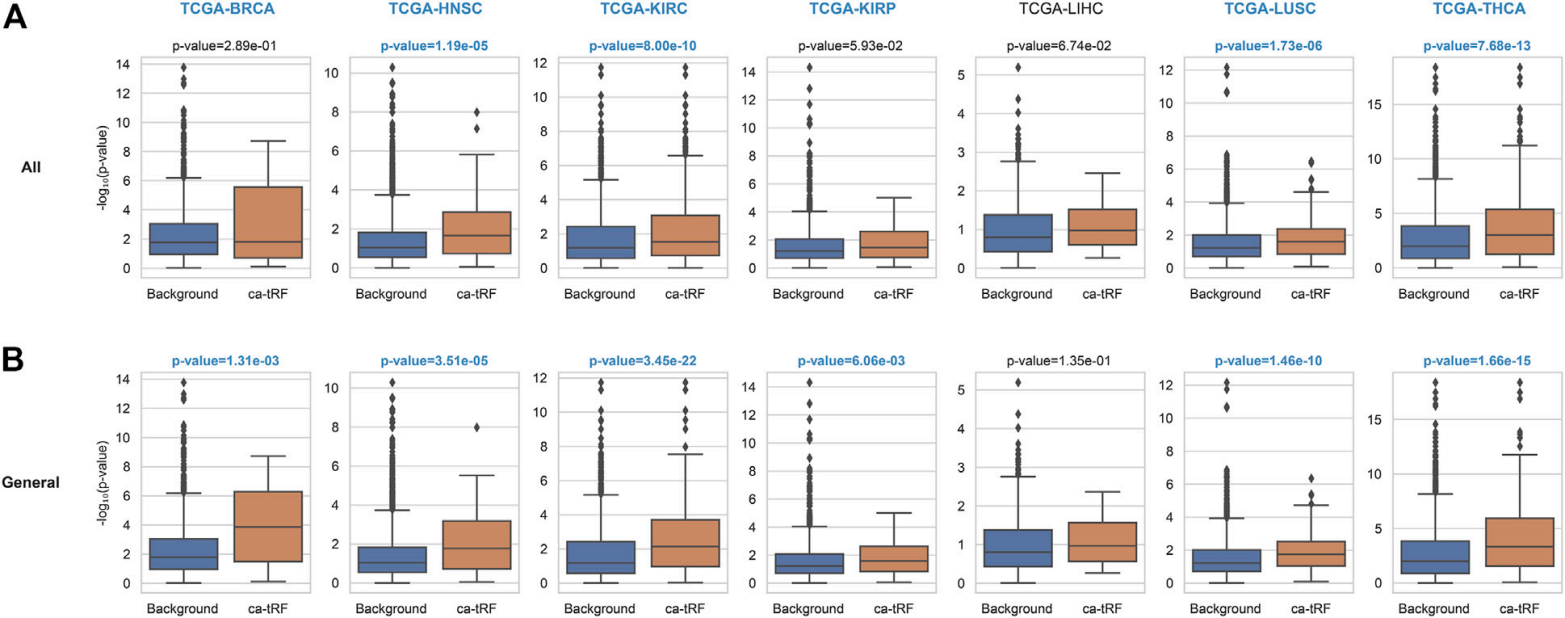
ca-tRFs are more likely to be correlated with T-cell infiltration. The correlation of tRF expression and T-cell infiltration degree are measured by Spearman’s correlation test. And the -log_10_ (*p*-value) distribution of **(A)** all ca-tRFs and **(B)** cancer-general ca-tRFs are respectively compared with background by Mann-Whitney test. The significant *p*-values (<0.05) and cancer names are shown with blue and bold font.

The functional enrichment results also revealed a few immuno-pathways and infectious processes like myeloid leukocyte activation, hepatitis, human T-cell leukemia virus 1 infection and bacterial invasion of epithelial cells, implying plausible immuno-regulatory roles of ca-tRFs. To further explore this point, we estimated the T-cell infiltration degree of tumor samples by gene expression deconvolution (See Materials and Methods) and further studied its correlation with ca-tRF expression abundance for each cancer type. As expected, ca-tRFs exhibit significantly higher correlations with T-cell infiltration than other tRFs (Figure 6). Considering the significance of T-cell infiltration in cancer prognosis (Gu-Trantien et al., 2013; Ge et al., 2019; Zhang et al., 2019), this result indicates the closer link between ca-tRFs and tumor immunity.

### ca-tRF-Based Cancer Subtypes are Informative to Prognosis

**FIGURE 7:**
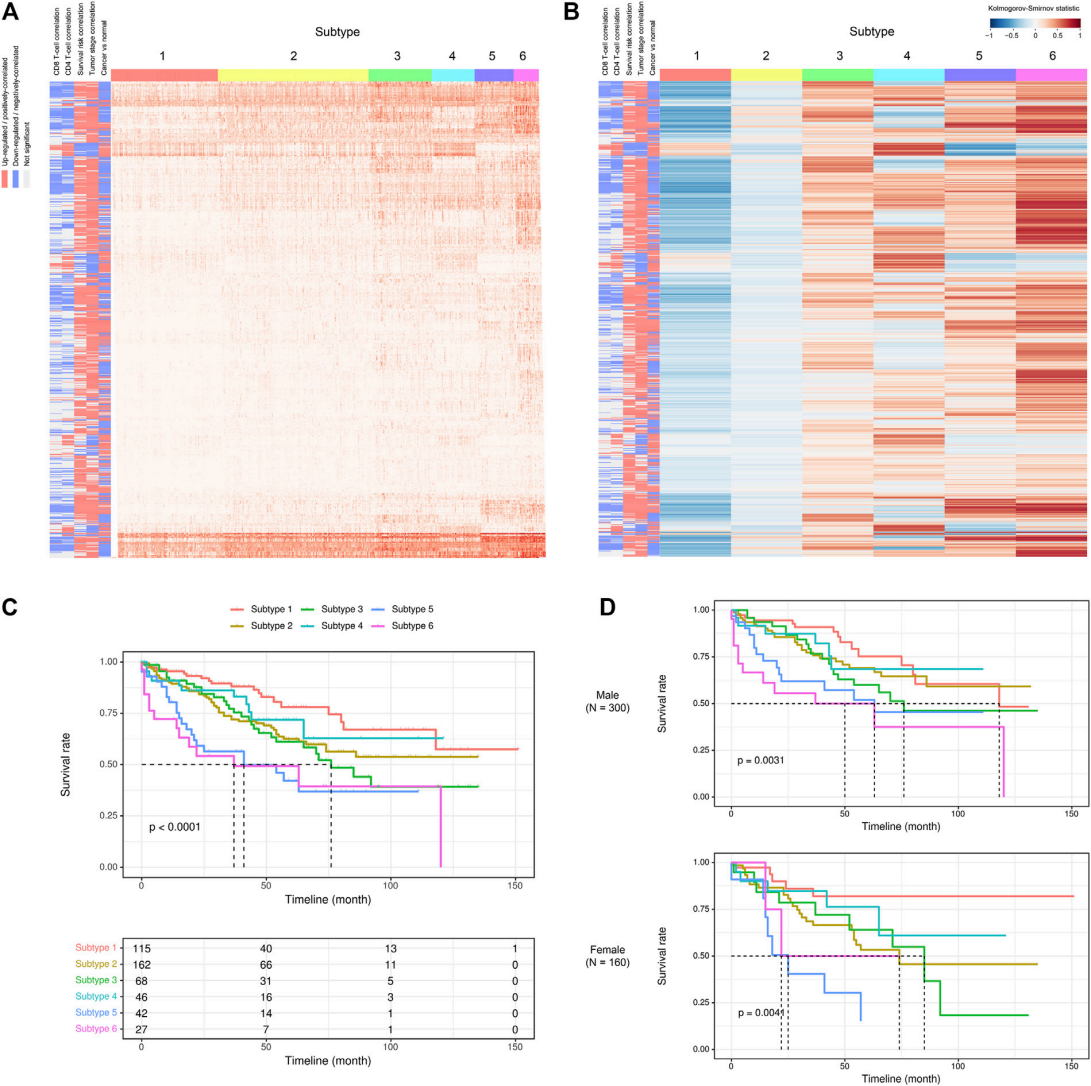
ca-tRF-based subtypes and corresponding survival distinction in KIRC. **(A)** The subtypes suggested by ca-tRF expression pattern. Columns and rows represent samples and ca-tRFs, respectively, and the color blocks along columns and rows represent subtypes and various ca-tRF-related biological features, respectively. **(B)** Heatmap of Kolmogorov-Smirnov statistics showing relative ca-tRF expression abundance across subtypes. **(C)** Kaplan-Meier plots showing the distinction of survival among different subtypes. The risk table showing the sample number at risk is also shown below for reference. **(D)** Sex-specific Kaplan-Meier plots showing the distinction of survival among different subtypes.

**FIGURE 8:**
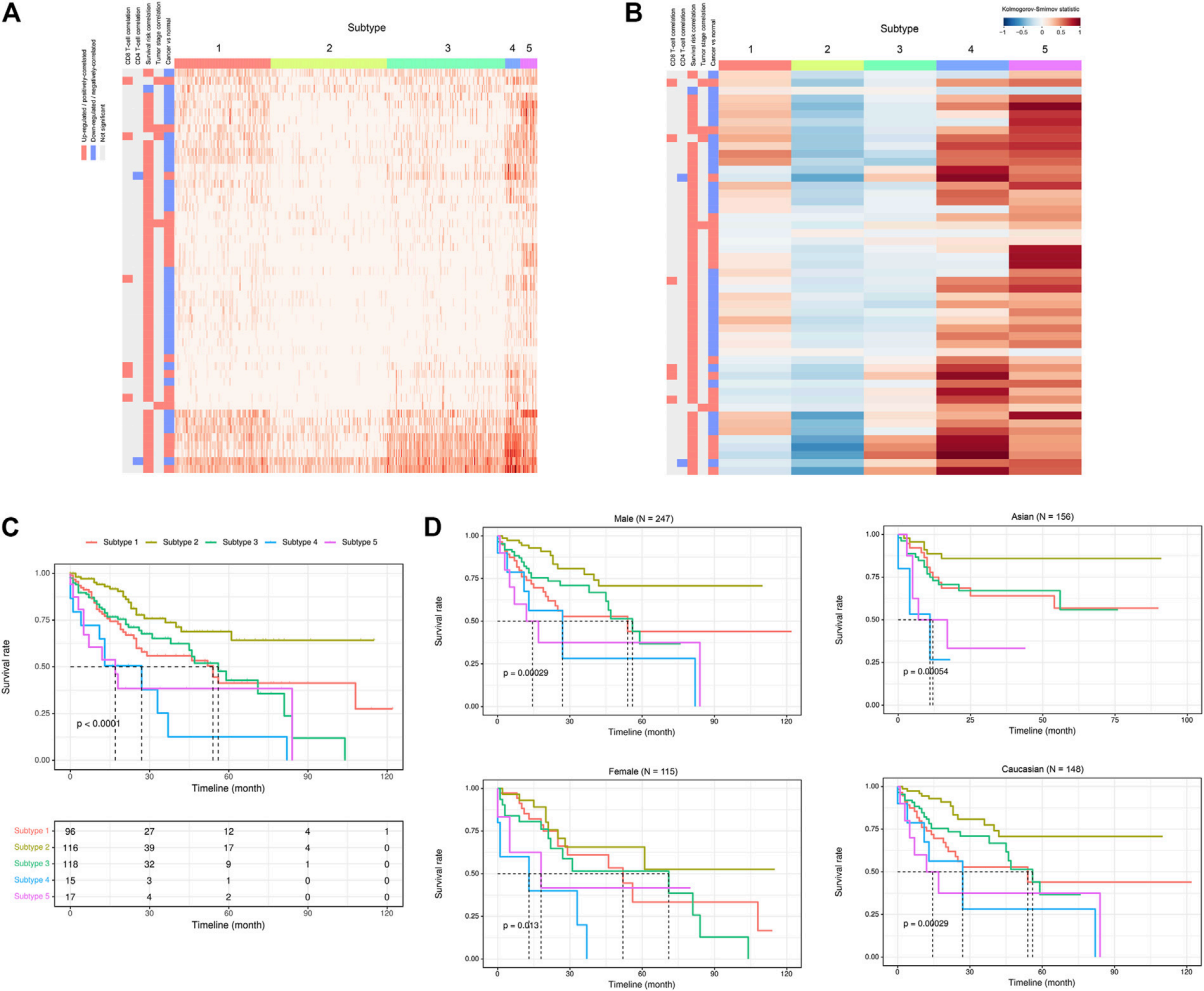
ca-tRF-based subtypes and corresponding survival distinction in LIHC. **(A)** The subtypes suggested by ca-tRF expression pattern. Columns and rows represent samples and ca-tRFs, respectively, and the color blocks along columns and rows represent subtypes and various ca-tRF-related biological features, respectively. **(B)** Heatmap of Kolmogorov-Smirnov statistics showing relative ca-tRF expression abundance across subtypes. **(C)** Kaplan-Meier plots showing the distinction of survival among different subtypes. The risk table showing the sample number at risk is also shown below for reference. **(D)** Sex- and race-specific Kaplan-Meier plots showing the distinction of survival among different subtypes.

Given the above-mentioned associations of ca-tRFs with cancer-related pathways and tumor immune infiltration, we further tested whether the ca-tRFs, if collectively investigated, could serve as an informative indicator of cancer prognosis. According to the ca-tRF expression profiles, we clustered the tumor samples under the consensus clustering framework (See Materials and Methods). Consequently, we identified five subtypes for HNSC, LIHC, and LUSC, six subtypes for KIRC, seven subtypes for BRCA and THCA and nine subtypes for KIRP (panels A–B of Figures 7, 8 and Supplementary Figures S2–S6; Supplementary Data S2). Among these cancers, HNSC, KIRC, KIRP, and LIHC exhibit significant survival differences among subtypes in overall, and the differences are especially obvious in KIRC and LIHC (*p*-value less than 0.0001) (panel C of Figures 7, 8 and Supplementary Figures S2–S6), suggesting the indicative role of ca-tRF-based subtyping in cancer prognosis. By contrast, the prognostic divergence is not significant in BRCA, LUSC, and THCA. Previous researches had reported the phenomenon of tRF expression disparity depending on sex and race (Telonis et al., 2015; Magee et al., 2018; Telonis and Rigoutsos, 2018; Magee and Rigoutsos, 2020). Inspired by this point, we further grouped the ca-tRF subtypes by sex and race (only the sex and race with sufficient samples considered) to investigate whether the disparity is also presented in survival rate (panel D of Figures 7, 8 and Supplementary Figures S3–S4). BRCA, LUSC, and THCA were not included in this analysis because their original ca-tRF subtypes are failed to show decent survival differences. As a result, the patterns of survival curves among different subtypes are still maintained when considering specific sex and race, indicating the robustness of ca-tRF subtyping.

### Comparison of ca-tRF-Based Subtypes With the External Subtypes

**FIGURE 9:**
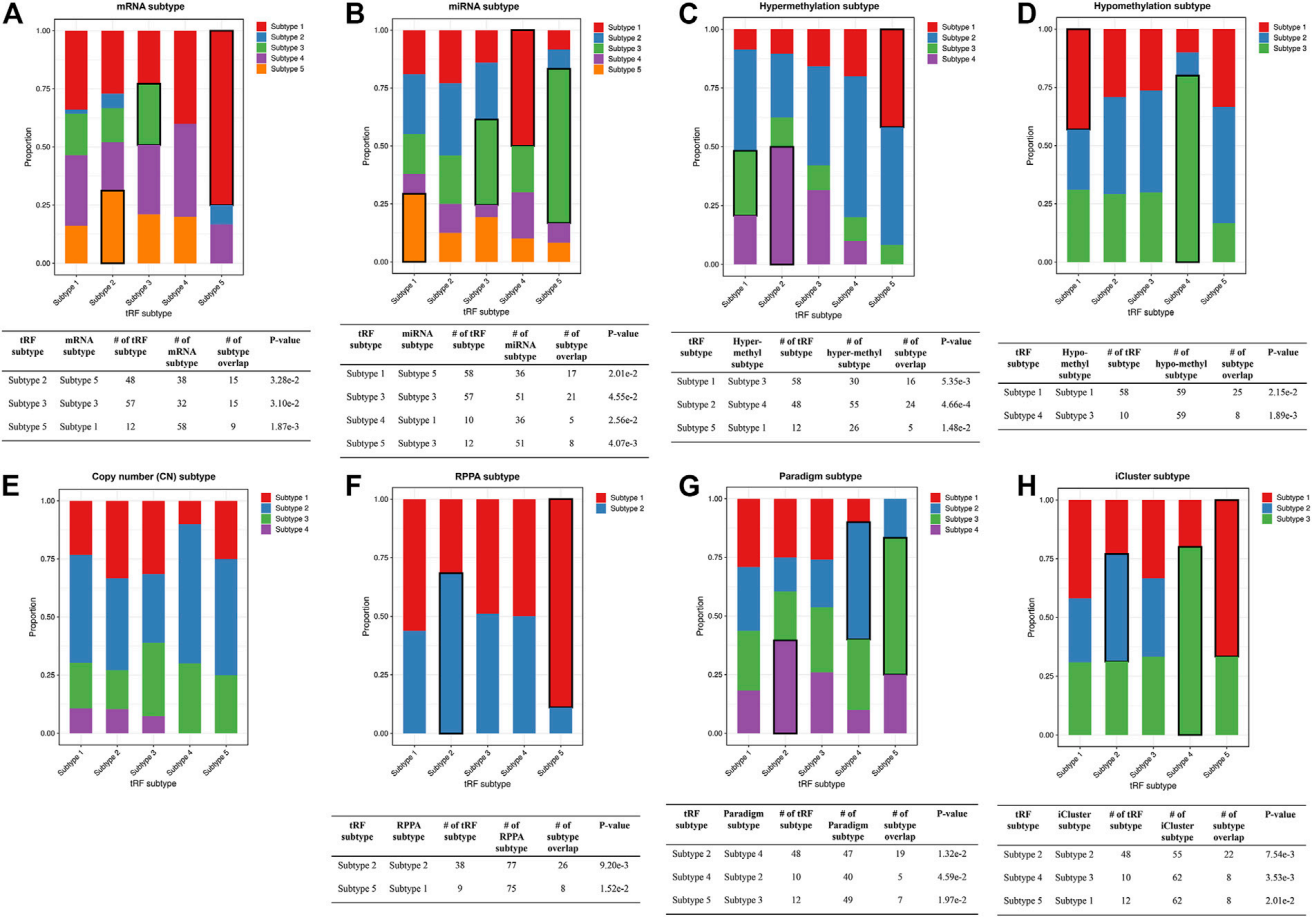
Stacked bar plots showing the relations between ca-tRF subtypes and external subtypes in LIHC. Stacked bar plots showing the enrichment of external subtypes (clustered by TCGA Consortia) in ca-tRF subtypes, including **(A)** mRNA subtypes identified by consensus clustering, **(B)** miRNA subtypes identified by non-negative matrix factorization, **(C–E)** hyper-methylation, hypo-methylation and SNP-based copy number subtypes identified by hierarchical clustering, **(F)** protein subtypes (namely, RPPA subtypes) identified by consensus clustering, **(G)** Paradigm subtypes and **(H)** multi-omics subtypes identified by iCluster. The statistical significance was measured by Fisher’s exact test with the *p*-value cutoff 0.05 and the significant relations are highlighted by black frames. The sample numbers of subtypes and subtype overlaps presented in the significant relations are also shown in the tables below.

Next, we further surveyed whether the ca-tRF-based subtypes could be linked or independent with the previous subtyping methods. In this comparison, only cancer types with sufficient external subtype data and showing significant prognostic divergence in ca-tRF-based subtyping were considered, containing LIHC (Figure 9), KIRC (Supplementary Figure S7) and KIRP (Supplementary Figure S8). For these three cancer types, various existing external subtypes based on mRNA, miRNA, methylation level and so on were covered in this comparison (Supplementary Data S4), including but not limited to the subtypes delineated by TCGA Consortia and identified by non-negative matrix factorization (Cancer Genome Atlas Research Network. Electronic address and Cancer Genome Atlas Research, 2017; Ricketts et al., 2018). We found most ca-tRF-based subtypes could decently overlap with at least one external subtype. However, as for subtype 2 of KIRC as well as subtype 2 and 6 of KIRP, no matched external subtype was found. Moreover, for all the surveyed cancers, although there are wide overlaps between ca-tRF-based subtypes and external subtypes, few ca-tRF-based subtypes could be fully represented by another external subtype. In other words, there are also detectable independence between ca-tRF subtypes and external subtypes, indicating tRFs’ potential to serve as novel prognostic factors that could supplement known subtyping.

To better demonstrate the associations and independence between the ca-tRF-based subtypes and the external subtypes, we further investigated LIHC subtypes in details because 1) ca-tRF-based subtypes show prominent survival differences in LIHC and 2) there are the largest number of external subtypes for reference in LIHC (8 kinds of subtypes in total), which could maximally reflect the relationships between the subtypes based on ca-tRFs and the other indicators. As depicted in Figure 8C, in LIHC, ca-tRF subtype 2 is of the best survival (low-risk group), ca-tRF subtypes 1 and 3 are with medium risks (medium-risk group) and ca-tRF subtypes 4 and 5 are the most malignant (high-risk group). Besides, the ca-tRF subtype 2, 4, and 5 significantly overlap with five, four, and six external subtypes, respectively. More importantly, the subtype 2, 4, and 5 were found significantly overlapped with iCluster subtypes (Figure 9H), which comprehensively integrate multi-dimensional data including transcriptome, miRNA expression pattern, methylome, proteome and genomic variation. Unlike ca-tRF subtype 2, 4, and 5 that show decent overlap with external subtypes, ca-tRF subtype 1 and 3 only match three and two external subtypes, respectively, and show no significant overlap with any iCluster subtypes, indicating they are relatively independent to the external subtypes (Figure 9).

**FIGURE 10:**
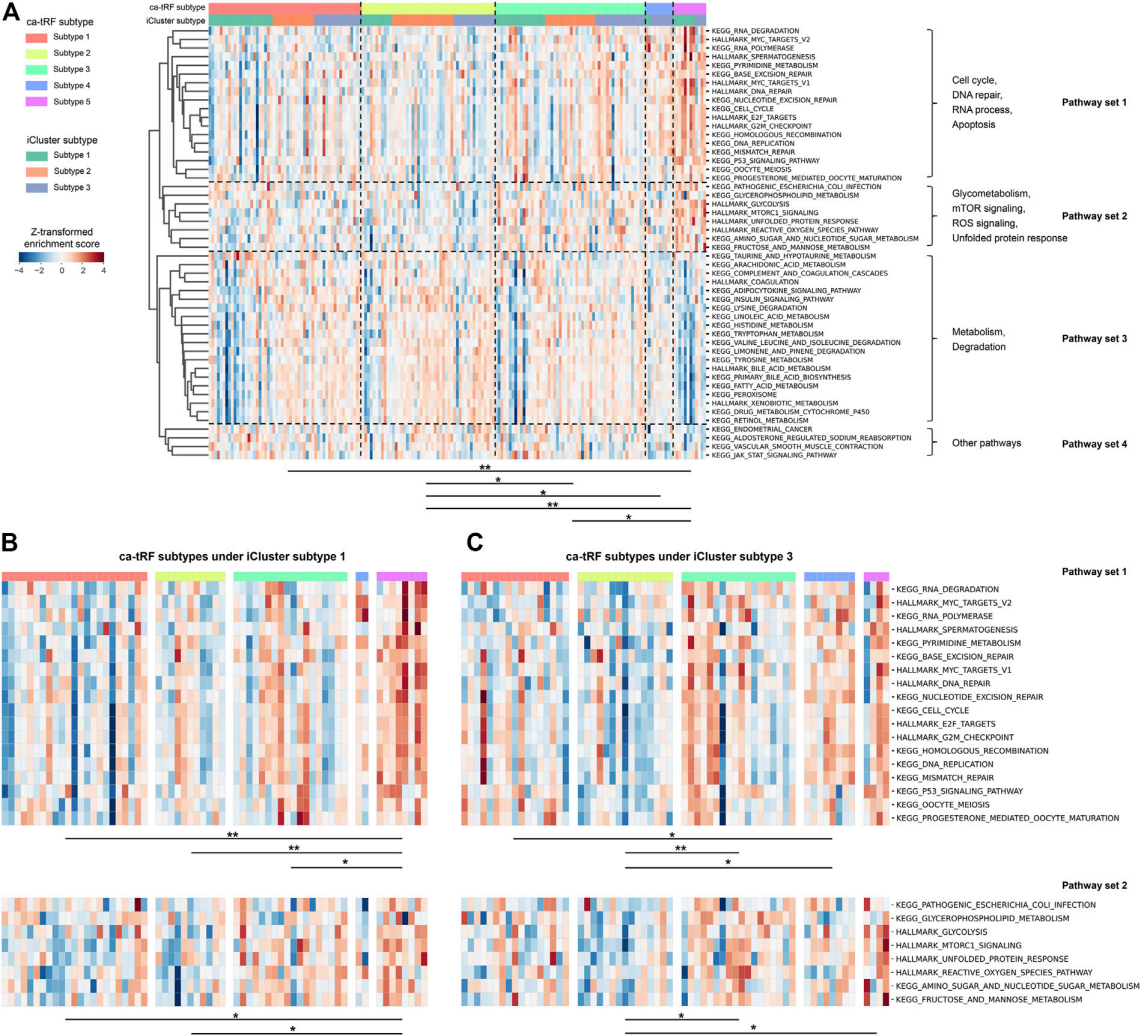
Distinct activities of biological pathways in LIHC revealed by ca-tRF-based subtyping regardless of the multi-omics characteristics-based iCluster subtypes 1 and 3. **(A)** Heatmap showing the enrichment scores (ES) of 50 most variable biological pathways among LIHC ca-tRF-based subtypes. The ca-tRF subtype and iCluster subtype assignments of each sample are indicated by the color stripes above the heatmap. The pathways could be clustered into four pathway sets, as annotated on the right side of the heatmap. **(B)** Heatmap showing the ESs of pathway set 1 and 2 among five ca-tRF subtypes under specific iCluster subtype 1. **(C)** Heatmap showing the ESs of pathway set 1 and 2 among five ca-tRF subtypes under specific iCluster subtype 3. For all panels, pairwise ES comparison results between ca-tRF subtypes are shown under the heatmap (Mann-Whitney test, * *p*-value < 0.05, ** *p*-value < 0.01).

We further noted that samples assigned to different ca-tRF-based subtypes would be associated with different pathways, even in the same iCluster subtype group. To compare the molecular signatures of different ca-tRF subtypes, we evaluated the activities of 236 biological pathways for LIHC samples (Supplementary Data S5) by ssGSEA enrichment scores (ES) and further screened 50 most variable pathways among ca-tRF subtypes by Kruskal-Wallis test (see Materials and Methods for more details). The screened pathways could be clustered into four pathway sets: the set 1 involves cell cycle, DNA repair, RNA process and apoptosis; the set 2 involves glycometabolism, mTOR signaling, ROS signaling and unfolded protein response; the set 3 includes many metabolism- and degradation-associated pathways; and the few other pathways were assigned to the pathway set 4 (Figure 10A). As expected, further pairwise comparisons demonstrate the pathway activities among ca-tRF subtypes are significantly different and meanwhile the low- and high-risk group exhibit more distinctive signatures than the medium-risk group (Figure 10A). More interestingly, we noted that the ES differences in pathway set 1 and 2 are still maintained when specifically considering iCluster subtype 1 (Figure 10B) and 3 (Figure 10C). That is to say, although the samples within the iCluster subtype 1 or 3 are of similar multi-omics characteristics, they will be distinguishable in many important pathway activities when further considering ca-tRF expression patterns. Such observation indicates that ca-tRF-based subtyping would specifically reveal the functional characteristics between different sample groups that are independent to the external multi-omics characteristics-based subtyping.

## Discussion

As a novel class of non-coding RNA, tRF has been getting increasing attention in recent years. Experimental researches have revealed that some tRFs could serve as biomarkers in some cancers (Falconi et al., 2019; Zhu et al., 2019). However, these low throughput experimental researches are not sufficient to explore the landscape of dysregulated tRFs at the pan-cancer level. In this work, according to TCGA samples’ tRF expression data provided by MINTbase v2.0, we screened ca-tRFs for seven TCGA cancer types with a “two out of three” strategy. From the pan-cancer perspective, the ca-tRFs are found significantly derived from the tRNA Val (TAC) and Arg (CCT). Moreover, we observed that most identified ca-tRFs are presented in multiple cancer types, and more interestingly, a considerable fraction of cancer-specific ca-tRFs are actually derived from the proximal region of cancer-general ca-tRFs on tRNAs. Unlike the biogenesis of canonical RNAs which are directly transcribed from the genome, tRFs are originated from the cleavage of mature tRNAs. Our results indicate the distribution of ca-tRFs on tRNAs are not random, and there are likely “hotspots” in tRNAs to produce ca-tRFs in tumors.

We also noticed that the variation tendency of ca-tRFs in three candidate tRF sets (i.e., cde-tRFs, tsca-tRFs, and psca-tRFs) are not always unidirectional. For example, a tRF may be significantly down-regulated in cancer samples in comparison with normal control but positively correlated with clinical tumor stage (Supplementary Data S1). This phenomenon demonstrates some tRFs may play opposite roles in oncogenesis and cancer progression. Meanwhile, it also reflects the advantages of our multi-view screening to capture comprehensive features of ca-tRFs.

Subsequent functional analysis reveals that the ca-tRF target gene modules participate in many oncogenesis and tumor progression-related processes such as ATF6-mediated unfolded protein response, angiogenesis, cell cycle process regulation, focal adhesion, PI3K-Akt signaling pathway, cellular senescence and FoxO signaling pathway. Meanwhile, the ca-tRFs also tend to be correlated with T-cell infiltration in comparison with other tRFs. Both results imply that ca-tRFs could play critical roles in cancer development and thus be of prognostic values. Indeed, cancer subtyping based on the ca-tRF expression pattern exhibit significant differences in survival and the differences are especially obvious in KIRC and LIHC. In addition, the survival patterns are still robust when considering specific sex and race, therefore ca-tRFs are potential to serve as universal prognostic factors. We also found most ca-tRF subtypes could decently link with external subtypes. But on the hand, the decent but not prominent overlaps also imply detectable independence between ca-tRF subtypes and external subtypes, indicating ca-tRFs would provide novel predictive clues for cancer prognosis. As a demonstration, we deeply investigated the relationships between ca-tRF subtypes and iCluster subtypes in LIHC, by means of ssGSEA. We found the ca-tRF expression patterns could associate with many pivotal biological pathways such as cell cycle, apoptosis, mTOR signaling pathway and so on regardless of the multi-omics characteristics of iCluster subtype 1 and 3, underlining the important regulatory roles of ca-tRFs.

Our analysis has systematically uncovered the potential roles of tRFs in oncogenesis and development and provided a reasonable ca-tRF list for future researches. However, there are still limitations in current work. The first limitation is in the step of investigating ca-tRF functions by the tRF-target interaction analysis, where we only considered the AGO-mediated tRF-gene interactions based on AGO-CLIP datasets. However, as we describe before, beyond binding with AGO-family proteins, tRFs also have various other functions which may involve some other RNA-binding proteins (RBP) (Couvillion et al., 2012; Kim et al., 2017). But to our best knowledge, so far there has not been tRF dataset focusing on the other RBPs because high-throughput techniques for profiling RBP interactions like CLIP-seq are mainly designed for mRNAs rather than tRFs. Suffering from this limitation, our results could only reveal partial ca-tRF functions in cancer. On the other hand, although there are evidences that the functions of some tRFs are indeed AGO-dependent, AGO-association presented in CLIP data do not necessarily indicate AGO-dependent mechanism-of-action. Therefore, in comparison with some technologies which can directly capture AGO-mediated small RNA-gene interactions (for example, CLASH) (Helwak et al., 2013), CLIP-derived interactions should contain more false positives. However, in the current stage, such CLASH-based datasets like are insufficient (usually identifying ∼1,000 tRF-gene pairs) to profile large-scale tRF-gene interactions. Therefore, we still adopted the more widely-used CLIP datasets, and to reduce false positives, we constrained the results with tight base pairing. In the previous work (Zhou et al., 2021), we validated the tRFs presented in the screened tRF-gene duplexes exhibit much better complementarily pairing abilities with CLIP-peaks than randomly generated small RNAs. Besides, the screened tRF-gene pairs are more prone to be co-expressed than background in TCGA samples, indicating the regulatory roles of these tRFs. Moreover, in accordance with the fact that AGO proteins are prone to bind smaller RNAs, ∼89% of the screened interactions involve tRFs less than 24 nt. When filtering the interactions with consistent co-expression in TCGA samples (i.e., the tRF-gene interaction set we used in this work), the ratio of interactions involving smaller tRFs will be dominating (∼98%). Overall, it could be rationally inferred that these interactions are with decent reliability. Another thing should be noted that Kumar et al. found the abundance of tRFs loaded on AGO2 are much lower than AGO1, AGO3 and AGO4 (Kumar et al., 2014), based on Hafner el al.’s HEK293 datasets (Hafner et al., 2010), plausibly indicating tRFs’ poor AGO2-binding ability. However, we found this phenomenon is not very repeatable in some other datasets such as Vongrad et al.’s human macrophage datasets (Vongrad et al., 2015), Benway et al.’s HK-2 datasets (Benway and Iacomini, 2018) and Hamilton et al.’s DU145 datasets (Hamilton et al., 2016). For example, in the AGO2-specific PAR-CLIP data of human macrophages generated by Vongrad et al., many tRFs are of hundreds of RPMs (median 150.13 RPMs of top 30 tRFs) and more surprisingly, much more abundant than miRNAs (median 5.80 RPMs of top 30 miRNAs). Higher abundances of tRFs relative to miRNAs were also observed in Benway et al.’s AGO2 PAR-CLIP data (median 32.86 RPMs of top 30 tRFs versus median 1.08 RPMs of top 30 miRNAs) and Hamilton et al.’s AGO2 PAR-CLIP data of DU145 cell line. What’s more, some publications have experimentally validated some tRFs should rely on AGO2 protein to perform downstream regulation (Li et al., 2012; Luo et al., 2018; Green et al., 2020). Therefore, the AGO2 protein is still considered in our pipeline.

The second limitation is due to the extreme scarcity of small RNA-sequencing data in public databases, we could not validate the ca-tRF-based subtyping in a totally independent dataset. Therefore, we could only perform validation on the original TCGA sample set. Specifically, for each cancer type, we randomly split the original set into training set and testing set with the ratio of 1:1, and then re-screened ca-tRFs on the training set and re-clustered tumor samples on both training and testing set based on the re-screened ca-tRFs. Subsequently, we measured the set similarity between the original ca-tRF subtypes and the re-clustered ca-tRF subtypes by Ochiai coefficient. As a result, the re-clustered subtypes exhibit decent consistency with the original subtypes, where most cancer types can reach at an Ochiai coefficient of 0.5 in average (Supplementary Figure S9). In other words, each original subtype could expectedly match with a highly overlapped re-clustered subtype, reflecting the robustness of ca-tRF-based subtyping. Nevertheless, the ca-tRF subtypes are still required to be further validated when the external resource of small RNA-sequencing is abundant enough in the future.

The third limitation worth discussing is, in this work we mainly surveyed tRF-cancer associations from the perspective of expression pattern. However, in recent years, many computational approaches based on machine learning and graphic theory are proposed to predict the associations between non-coding RNAs and diseases (Chen et al., 2018; Zeng et al., 2018), which provides another approach to discover novel tRF-cancer associations. However, an obligate perquisite of these algorithms is a sizable training dataset derived from prior experimental knowledge. And to our best knowledge, existing publications about tRF and cancer are extremely scarce (actually less than 200, by querying the keywords “tsRNA cancer” and “tRNA-derived fragment cancer” in PubMed). Therefore, in current stage, existing data have not been able to support these algorithms, but we believe it would be feasible with the accumulation of experimental data in the future.

## Data Availability

The original contributions presented in the study are included in the article/Supplementary Material, further inquiries can be directed to the corresponding author.
