# Supplementary material for: Screening and Comprehensive Analysis of Cancer-Associated tRNA-Derived Fragments
Source: Front Genet. 2022 Jan 14;12:747931. doi: 10.3389/fgene.2021.747931 (PMC8795687; doi:10.3389/fgene.2021.747931)
Supplement: Supplementary file 6 [file Image1.PDF]

## Supplementary Figures

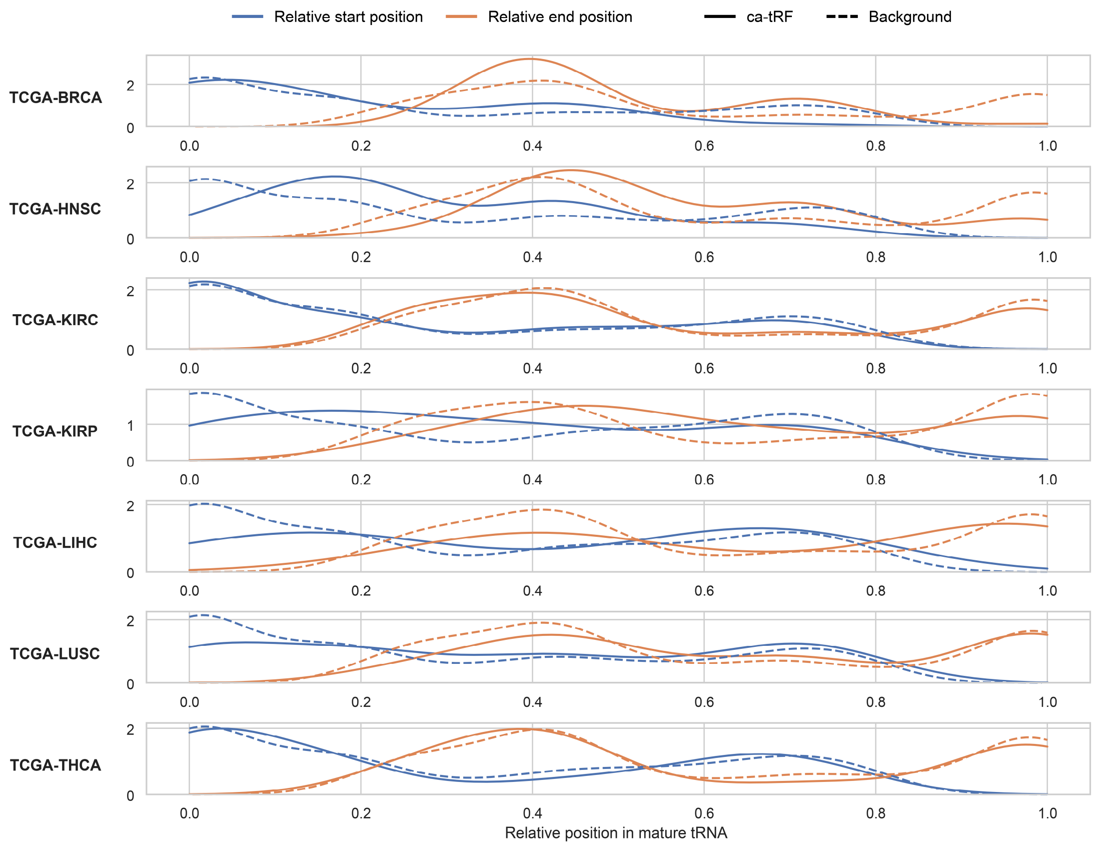


**Figure S1. Relative positions of ca-tRFs in mature tRNA.**

The probability density plot of relative start/end positions of ca-tRFs/background in mature tRNA is shown.

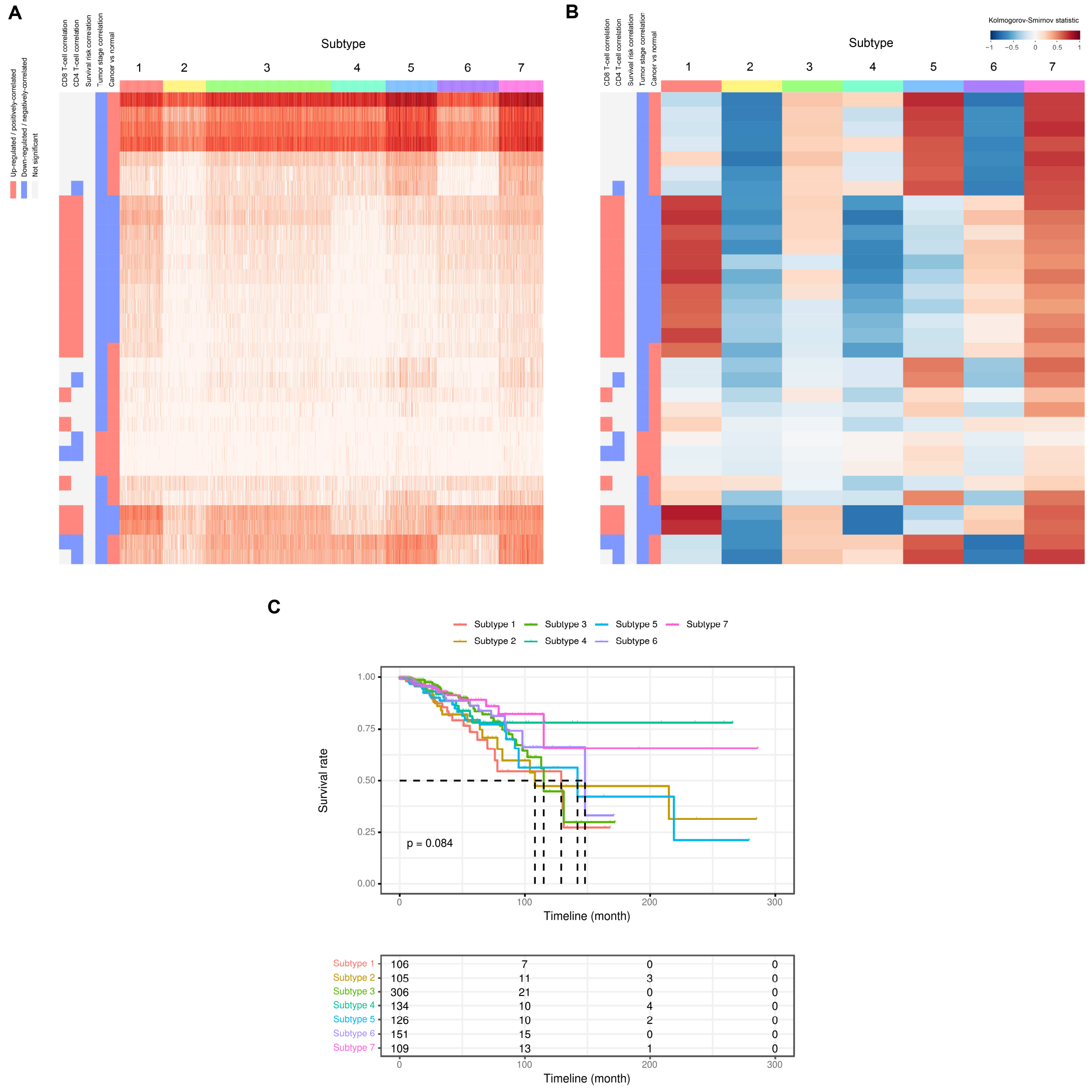


**Figure S2. ca-tRF-based subtypes and corresponding survival distinction in BRCA.**

(A) The subtypes suggested by ca-tRF expression pattern. Columns and rows represent samples and ca-tRFs, respectively, and the color blocks along columns and rows represent subtypes and various ca-tRF-related biological features, respectively. (B) Heatmap of Kolmogorov-Smirnov statistics showing relative ca-tRF expression abundance across subtypes. (C) Kaplan-Meier plots showing the distinction of survival among different subtypes. The risk table showing the sample number at risk is also shown below for reference.

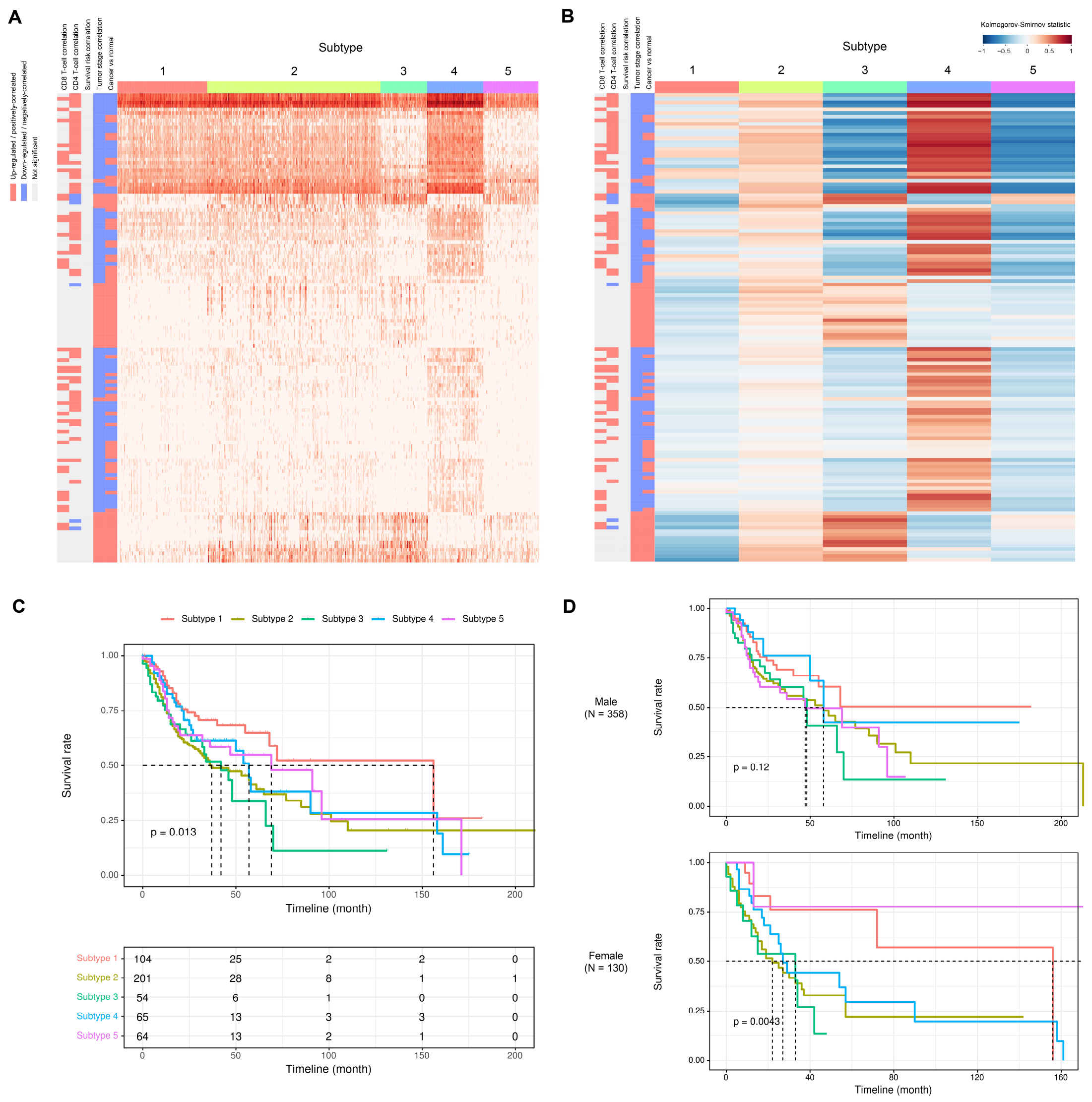


**Figure S3. ca-tRF-based subtypes and corresponding survival distinction in HNSC.**

(A) The subtypes suggested by ca-tRF expression pattern. Columns and rows represent samples and ca-tRFs, respectively, and the color blocks along columns and rows represent subtypes and various ca-tRF-related biological features, respectively. (B) Heatmap of Kolmogorov-Smirnov statistics showing relative ca-tRF expression abundance across subtypes. (C) Kaplan-Meier plots showing the distinction of survival among different subtypes. The risk table showing the sample number at risk is also shown below for reference. (D) Sex-specific Kaplan-Meier plots showing the distinction of survival among different subtypes.

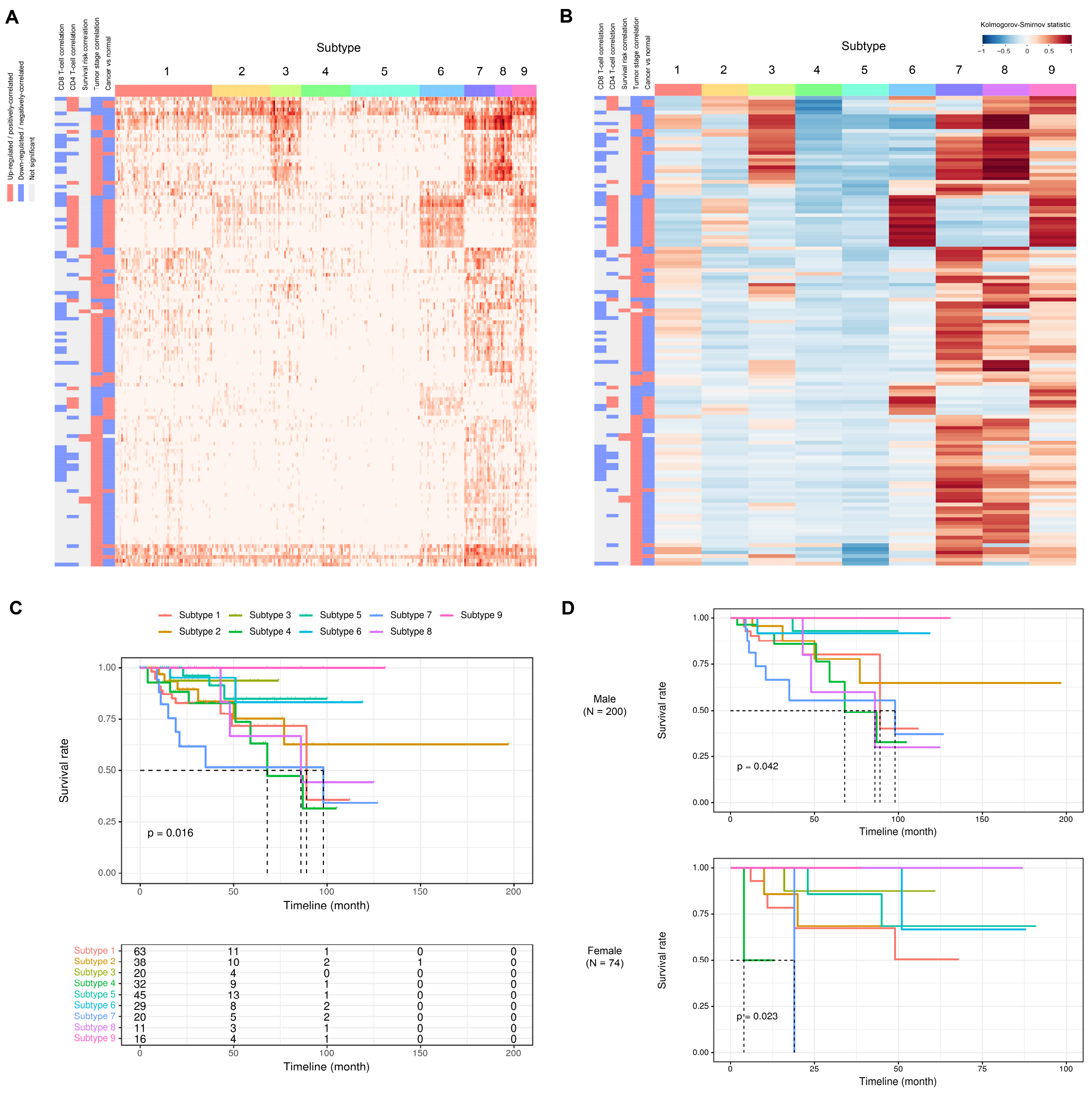


**Figure S4. ca-tRF-based subtypes and corresponding survival distinction in KIRP.**

(A) The subtypes suggested by ca-tRF expression pattern. Columns and rows represent samples and ca-tRFs, respectively, and the color blocks along columns and rows represent subtypes and various ca-tRF-related biological features, respectively. (B) Heatmap of Kolmogorov-Smirnov statistics showing relative ca-tRF expression abundance across subtypes. (C) Kaplan-Meier plots showing the distinction of survival among different subtypes. The risk table showing the sample number at risk is also shown below for reference. (D) Sex-specific Kaplan-Meier plots showing the distinction of survival among different subtypes.

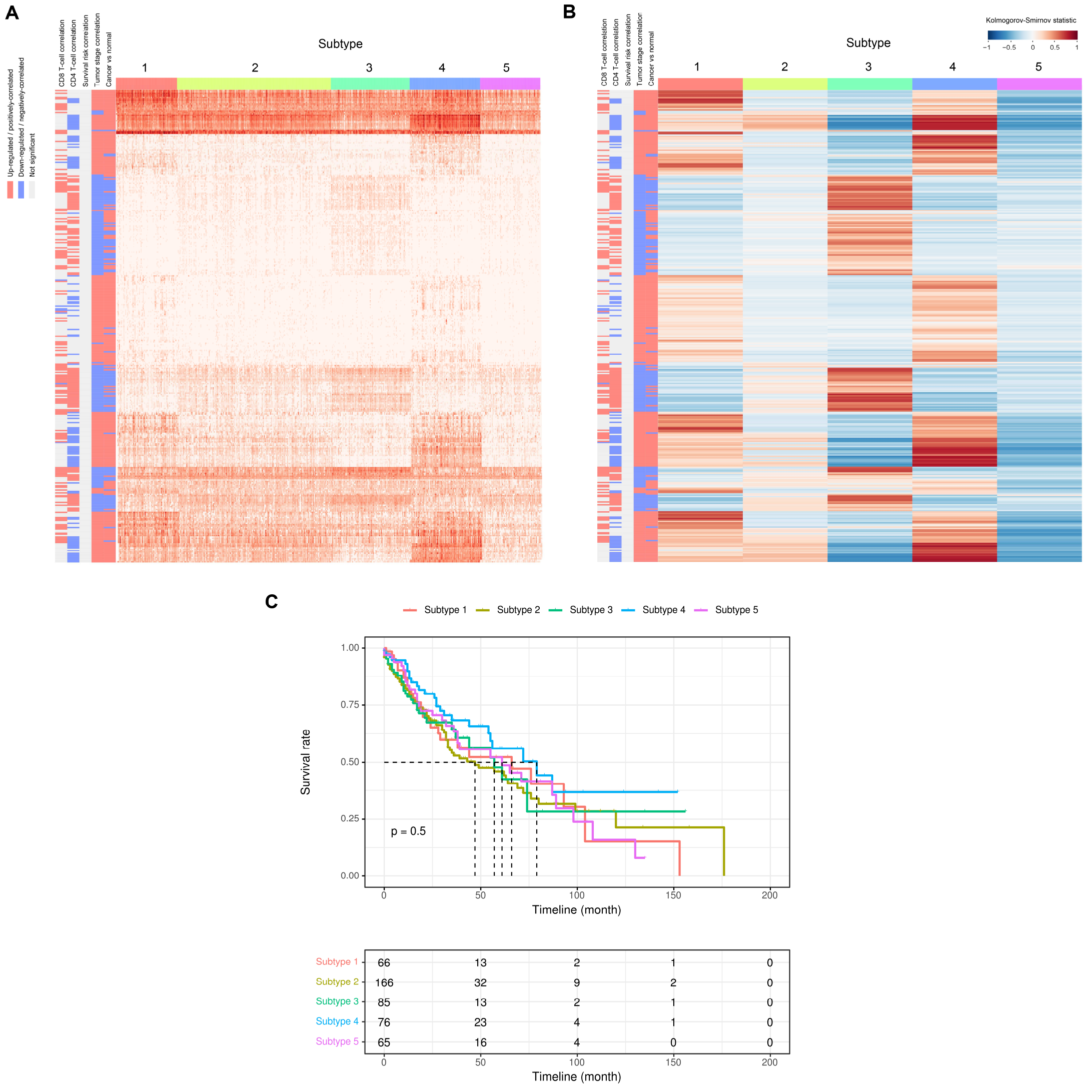


**Figure S5. ca-tRF-based subtypes and corresponding survival distinction in LUSC.**

(A) The subtypes suggested by ca-tRF expression pattern. Columns and rows represent samples and ca-tRFs, respectively, and the color blocks along columns and rows represent subtypes and various ca-tRF-related biological features, respectively. (B) Heatmap of Kolmogorov-Smirnov statistics showing relative ca-tRF expression abundance across subtypes. (C) Kaplan-Meier plots showing the distinction of survival between different subtypes. The risk table showing the sample number at risk is also shown below for reference.

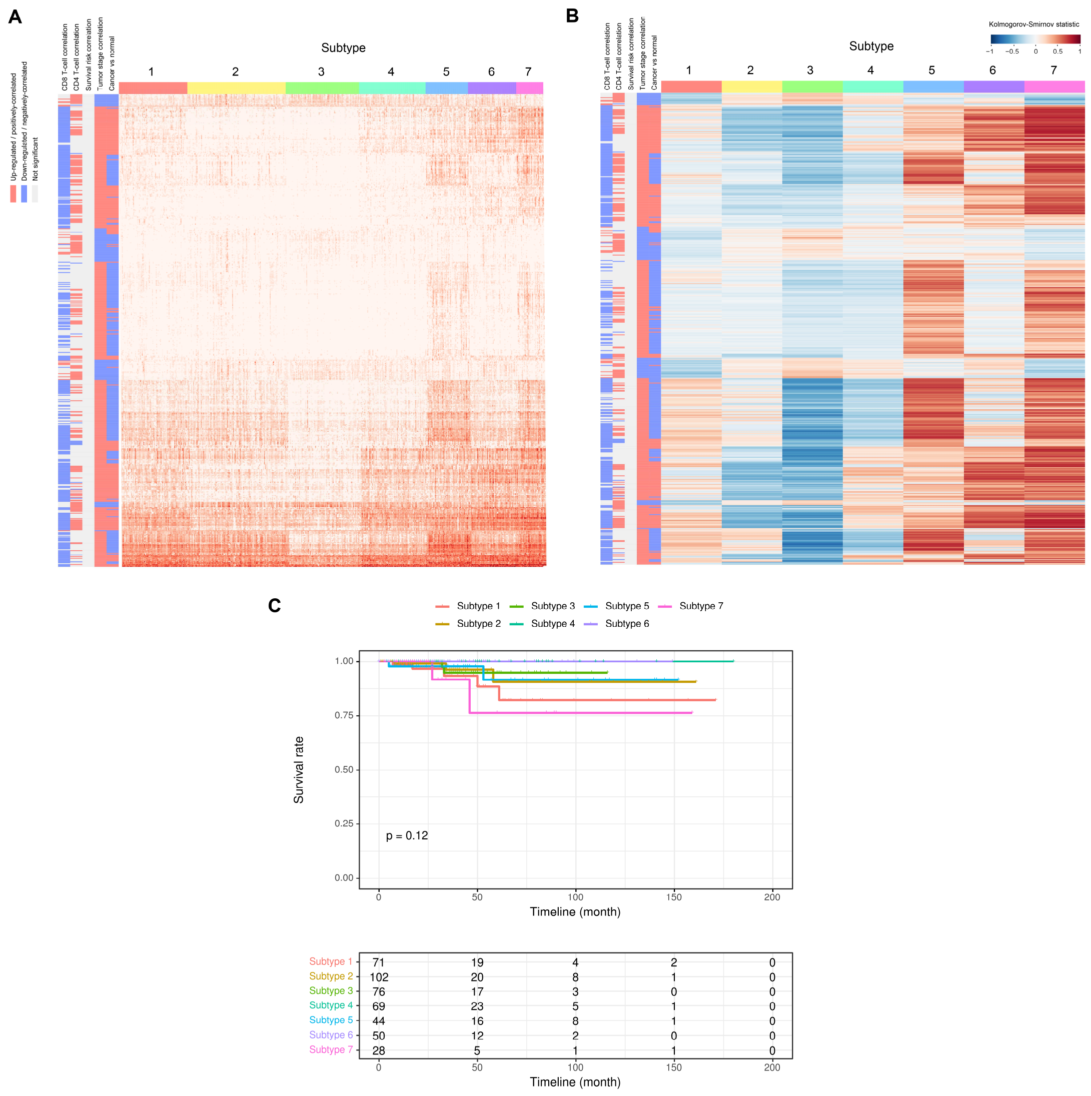


**Figure S6. ca-tRF-based subtypes and corresponding survival distinction in THCA.**

(A) The subtypes suggested by ca-tRF expression pattern. Columns and rows represent samples and ca-tRFs, respectively, and the color blocks along columns and rows represent subtypes and various ca-tRF-related biological features, respectively. (B) Heatmap of Kolmogorov-Smirnov statistics showing relative ca-tRF expression abundance across subtypes. (C) Kaplan-Meier plots showing the distinction of survival between different subtypes. The risk table showing the sample number at risk is also shown below for reference.

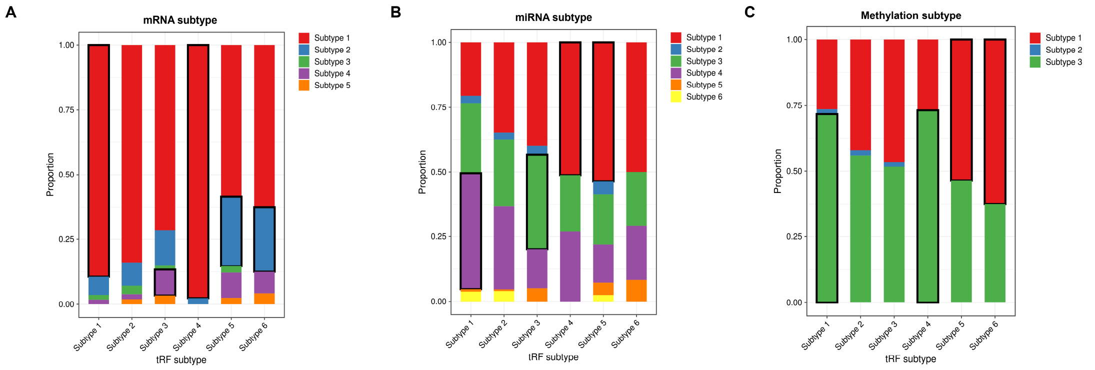


| tRF subtype | mRNA subtype | # of tRF subtype | # of mRNA subtype | # of subtype overlap | P-value |
|---|---|---|---|---|---|
| Subtype 1 | Subtype 1 | 113 | 356 | 101 | 6.92e-3 |
| Subtype 3 | Subtype 4 | 60 | 17 | 6 | 1.90e-2 |
| Subtype 4 | Subtype 1 | 43 | 356 | 42 | 1.08e-3 |
| Subtype 5 | Subtype 2 | 41 | 48 | 11 | 2.29e-3 |
| Subtype 6 | Subtype 2 | 24 | 48 | 6 | 3.69e-2 |

| tRF subtype | miRNA subtype | # of tRF subtype | # of miRNA subtype | # of subtype overlap | P-value |
|---|---|---|---|---|---|
| Subtype 1 | Subtype 4 | 107 | 128 | 48 | 1.32e-4 |
| Subtype 3 | Subtype 3 | 60 | 112 | 22 | 3.87e-2 |
| Subtype 4 | Subtype 1 | 41 | 154 | 21 | 2.84e-2 |
| Subtype 5 | Subtype 1 | 41 | 154 | 22 | 1.26e-2 |

| tRF subtype | methyl subtype | # of tRF subtype | # of methyl subtype | # of subtype overlap | P-value |
|---|---|---|---|---|---|
| Subtype 1 | Subtype 3 | 106 | 250 | 76 | 1.33e-3 |
| Subtype 4 | Subtype 3 | 41 | 250 | 30 | 3.56e-2 |
| Subtype 5 | Subtype 1 | 41 | 168 | 22 | 3.99e-2 |
| Subtype 6 | Subtype 1 | 24 | 168 | 15 | 1.69e-2 |

**Figure S7. Stacked bar plots showing the relations between ca-tRF subtypes and external subtypes in KIRC.**

Stacked bar plots showing the enrichment of external subtypes (clustered by Ricketts et al.) in ca-tRF subtypes, including (A-B) mRNA and miRNA subtypes identified by consensus clustering and (C) methylation subtypes identified hierarchical clustering. The statistical significance was measured by Fisher's exact test with the p-value cutoff 0.05 and the significant relations are highlighted by black frames. The sample numbers of subtypes and subtype overlaps presented in the significant relations are also shown in the tables below.

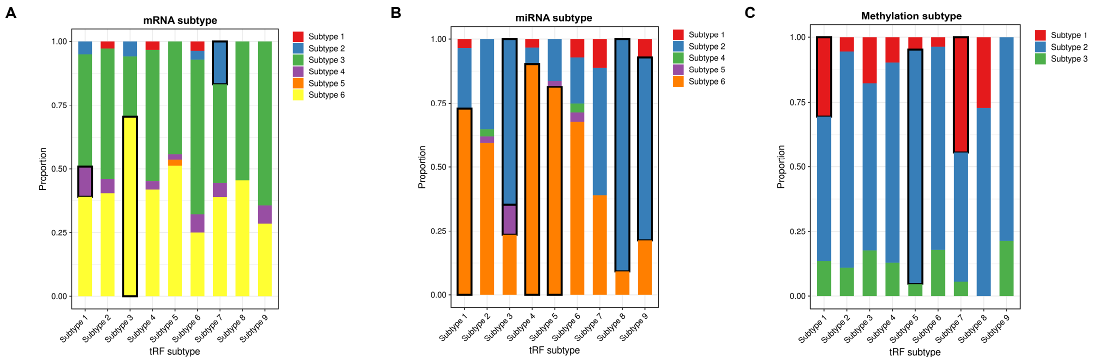


| tRF subtype | mRNA subtype | # of tRF subtype | # of mRNA subtype | # of subtype overlap | P-value |
|---|---|---|---|---|---|
| Subtype 1 | Subtype 4 | 59 | 18 | 7 | 3.20e-2 |
| Subtype 3 | Subtype 6 | 17 | 108 | 12 | 1.32e-2 |
| Subtype 7 | Subtype 2 | 18 | 8 | 3 | 1.29e-2 |

| tRF subtype | miRNA subtype | # of tRF subtype | # of miRNA subtype | # of subtype overlap | P-value |
|---|---|---|---|---|---|
| Subtype 1 | Subtype 6 | 59 | 162 | 43 | 4.56e-2 |
| Subtype 3 | Subtype 2 | 17 | 81 | 11 | 3.61e-3 |
| Subtype 3 | Subtype 5 | 17 | 5 | 2 | 3.64e-2 |
| Subtype 4 | Subtype 6 | 31 | 162 | 28 | 3.14e-4 |
| Subtype 5 | Subtype 6 | 43 | 162 | 35 | 3.67e-3 |
| Subtype 8 | Subtype 2 | 11 | 81 | 10 | 5.06e-5 |
| Subtype 9 | Subtype 2 | 14 | 81 | 10 | 1.86e-3 |

| tRF subtype | methyl subtype | # of tRF subtype | # of methyl subtype | # of subtype overlap | P-value |
|---|---|---|---|---|---|
| Subtype 1 | Subtype 1 | 57 | 40 | 18 | 5.93e-4 |
| Subtype 5 | Subtype 2 | 43 | 188 | 39 | 1.98e-3 |
| Subtype 7 | Subtype 1 | 18 | 40 | 8 | 2.28e-3 |

**Figure S8. Stacked bar plots showing the relations between ca-tRF subtypes and external subtypes in KIRP.**

Stacked bar plots showing the enrichment of external subtypes (clustered by Ricketts et al.) in ca-tRF subtypes, including (A-B) mRNA and miRNA subtypes identified by consensus clustering and (C) methylation subtypes identified hierarchical clustering. The statistical significance was measured by Fisher's exact test with the p-value cutoff 0.05 and the significant relations are highlighted by black frames. The sample numbers of subtypes and subtype overlaps presented in the significant relations are also shown in the tables below.

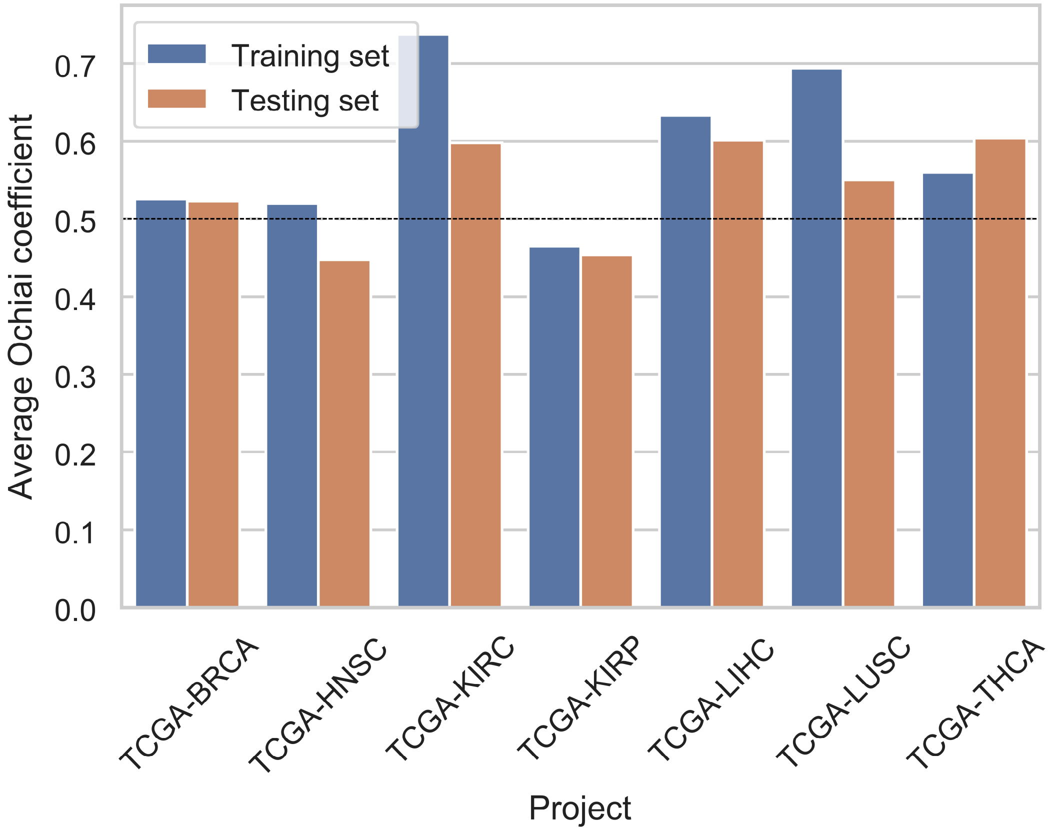


**Figure S9. Average Ochiai coefficient illustrating robustness of ca-tRF-based subtyping.** After randomly splitting the original set into training set and testing set with the ratio of 1:1, we re-screened ca-tRFs on the training set and then re-clustered tumor samples on both training and testing set based on the re-screened ca-tRFs. The average Ochiai coefficient of each cancer type between the original ca-tRF subtypes and the re-clustered ca-tRF subtypes are depicted in the bar plot, where the dashed line represents the Ochiai coefficient of 0.5.
